# Hyperoside protects against poly-GR-mediated neurodegeneration via regulation of mitochondrial fission and oxidative stress in *C9orf72*-associated ALS

**DOI:** 10.1186/s13020-026-01433-w

**Published:** 2026-06-04

**Authors:** Wen-Chi Hsieh, Chun-Yu Lin, Hsuan-Cheng Wu, Eddie Feng-Ju Weng, Shao-Ming Wang

**Affiliations:** 1 Neuroscience and Brain Disease Center, China Medical University, Taichung, 404328 Taiwan; 2 Graduate Institute of Biomedical Sciences, College of Medicine, China Medical University, Taichung, 404328 Taiwan; 3 School of Medicine, College of Medicine, China Medical University, Taichung, 404328 Taiwan

**Keywords:** Hyperoside, *C9orf72*-associated ALS, Mitochondrial dysfunction, Oxidative stress, Nrf2

## Abstract

**Background:**

Arginine-rich poly-glycine-arginine (poly-GR), a toxic dipeptide repeat protein generated from *C9orf72* hexanucleotide repeat expansion, drives mitochondrial dysfunction, oxidative stress, and neuronal loss in amyotrophic lateral sclerosis (ALS). Hyperoside, a bioactive flavonoid, exhibits antioxidant and cytoprotective properties, but its therapeutic relevance to *C9orf72*-associated ALS remains unclear.

**Purpose:**

To determine whether hyperoside attenuates poly-GR-induced mitochondrial and oxidative injury and improves neuronal survival in cellular and animal models of *C9orf72*-ALS.

**Methods:**

A combined in vitro and in vivo experimental study using motor neuron-like cells and an AAV-mediated neonatal mouse model of poly-GR toxicity. NSC34 cells expressing EGFP-GR_50_ were analyzed for mitochondrial morphology, membrane potential, ROS generation, antioxidant signaling, and apoptosis using confocal microscopy, CellROX/MitoTracker assays, Western blot analysis, and viability testing. For in vivo assessment, neonatal mice received intracerebroventricular AAV9-EGFP-GR50 followed by intraperitoneal hyperoside (10 mg/kg). Survival, cerebral hemisphere length, and cortical NeuN⁺ neuron numbers were quantified.

**Results:**

Poly-GR expression induced pronounced mitochondrial fragmentation, reduced membrane potential, elevated ROS, and suppressed Nrf2/HO-1/GPx4 signaling, accompanied by increased Drp1 and reduced Opa1 expression. Hyperoside reversed these abnormalities by restoring mitochondrial integrity, normalizing the Drp1/Opa1 balance, enhancing Nrf2 nuclear accumulation, and increasing the expression of HO-1 and GPx4. Hyperoside also reduced cleaved caspase-3 and corrected the Bax/Bcl-2 ratio, improving cell viability under basal and oxidative stress conditions. In vivo, hyperoside modestly prolonged survival, increased cerebral hemisphere length, and significantly preserved cortical neuronal numbers in AAV9-EGFP-GR_50_ mice.

**Conclusion:**

Hyperoside mitigates poly-GR-induced neurotoxicity by alleviating excessive mitochondrial fission, strengthening Nrf2-dependent antioxidant defenses, and suppressing apoptosis. These findings support hyperoside as a promising multi-target therapeutic candidate for *C9orf72*-associated ALS.

**Graphical abstract:**

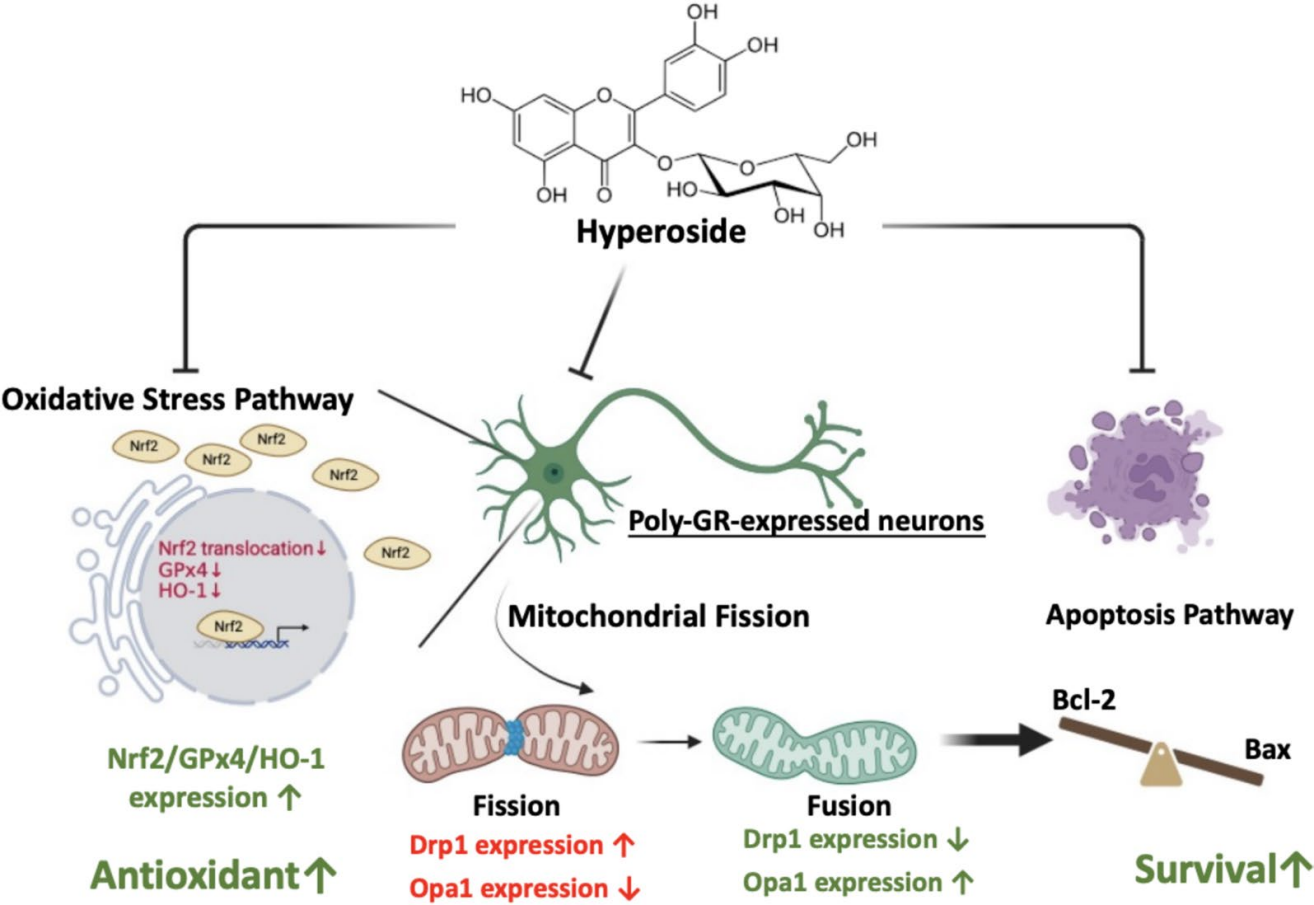

**Supplementary Information:**

The online version contains supplementary material available at 10.1186/s13020-026-01433-w.

## Highlights

- Poly-GR induces mitochondrial fragmentation and oxidative stress in neurons.
- Hyperoside rescues mitochondrial morphology.
- Hyperoside activates Nrf2 signaling, enhancing HO-1 and GPx4 antioxidant defenses.
- Hyperoside reduces apoptosis and preserves mitochondrial membrane potential.
- In vivo hyperoside improves survival and neuronal numbers in EGFP-GR_50_ mice.
- Hyperoside as a multi-target therapeutic candidate for *C9orf72*-ALS.

**Supplementary Information:**

The online version contains supplementary material available at 10.1186/s13020-026-01433-w.

## Introduction

Amyotrophic lateral sclerosis (ALS) is a fatal neurodegenerative disease marked by progressive loss of upper and lower motor neurons, ultimately causing limb weakness, bulbar impairment, and respiratory failure [1, 2]. Although major advances have been made in understanding ALS pathology, no curative therapy exists, and the average survival remains only 3–5 years after symptom onset. Among known genetic contributors, the C9orf72 hexanucleotide repeat expansion is the most prevalent cause of both familial and sporadic ALS [1, 3]. This mutation generates toxic G4C2 repeat RNA and five repeat-associated non-AUG (RAN)-translated dipeptide repeat proteins (DPRs) [4, 5]. The arginine-rich DPRs, particularly poly-GR, exhibit pronounced neurotoxicity and are strongly implicated in the degeneration of motor neurons [6–8]. Poly-GR preferentially accumulates in ALS-vulnerable regions, including the motor cortex and the anterior horn of the spinal cord, highlighting its central role in disease pathogenesis [9]. Accordingly, this study focuses on poly-GR-induced neuronal toxicity.

Multiple mechanisms contribute to poly-GR-induced neurotoxicity, with mitochondrial dysfunction and redox imbalance being particularly prominent [8, 10]. In *C9orf72*-ALS, excessive ROS arising from impaired mitochondrial activity, NOX activation, or weakened antioxidant defenses drives lipid peroxidation and neuronal degeneration [10–12]. The Nrf2 pathway serves as a central regulator of cellular redox homeostasis, activating a battery of antioxidant genes upon stimulation [13, 14]. Poly-GR toxicity is also linked to disrupted mitochondrial dynamics [8]; however, the precise mechanisms by which poly-GR alters these processes remain unclear. These gaps underscore the need to identify natural compounds that can restore Nrf2 signaling and mitochondrial homeostasis in poly-GR-induced *C9orf72*-ALS.

Hyperoside (quercetin-3-O-*β*-D-galactopyranoside) is a bioactive flavonoid glycoside and a major constituent of Crataegus pinnatifida Bunge [15, 16]. It possesses diverse pharmacological properties, including antioxidant, anti-inflammatory, antidepressant, and neuroprotective effects [16–19]. Hyperoside has been shown to ameliorate cognitive deficits and mitochondrial dysfunction in Alzheimer’s disease models [19], protect dopaminergic neurons in Parkinson’s disease models [20], and suppress oxidative stress-related cellular injury [21]. Its regulatory effects on mitochondrial dynamics and its ability to activate the Nrf2 pathway further suggest therapeutic potential in neurodegenerative diseases. However, the potential therapeutic efficacy of hyperoside in ALS, particularly in *C9orf72*-related poly-GR toxicity, has not yet been investigated.

**Fig. 1:**
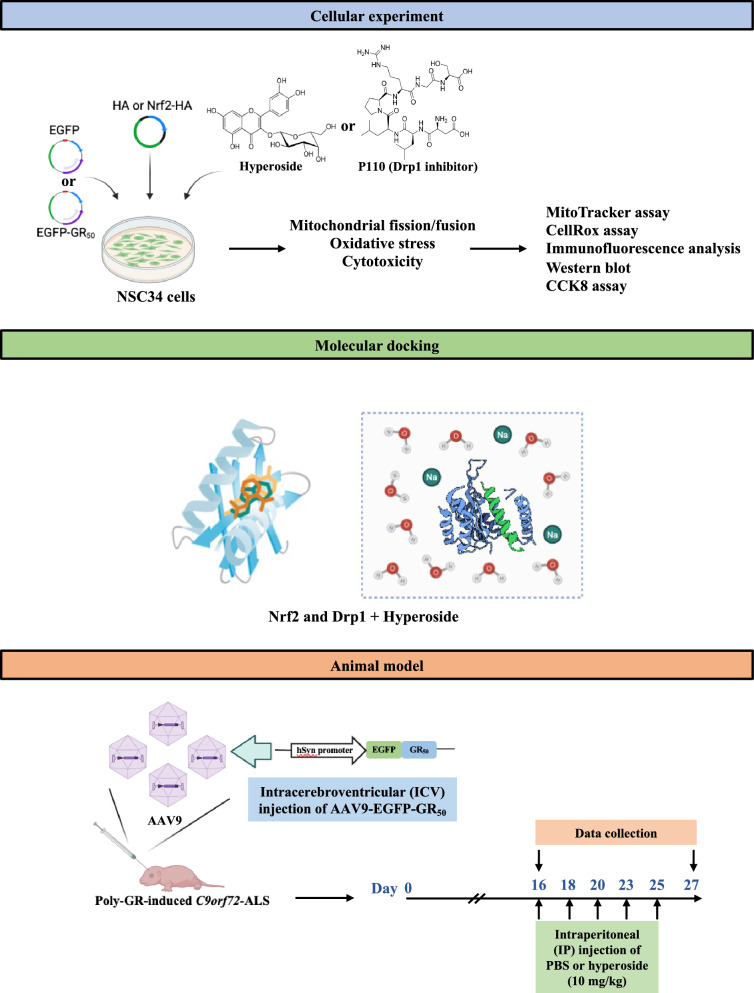
Schematic overview of the experimental design. The study design includes cellular experiments, molecular docking analysis, and an in vivo mouse model. (Upper panel) NSC34 cells were transfected with EGFP or EGFP-GR₅₀, or co-transfected with Nrf2-HA as indicated, followed by treatment with hyperoside or P110 (a selective Drp1 inhibitor). Mitochondrial fission/fusion, oxidative stress, and cytotoxicity were evaluated using MitoTracker staining, CellROX assay, immunofluorescence, Western blotting, and CCK-8 assays. (Middle panel) In silico molecular docking analysis was performed to predict potential binding interactions between hyperoside and Nrf2 or Drp1. (Lower panel) For in vivo studies, AAV9-hSyn-EGFP-GR₅₀ was delivered via intracerebroventricular (ICV) injection to establish a poly-GR-induced *C9orf72*-associated ALS mouse model. Mice received intraperitoneal (IP) injections of PBS or hyperoside (10 mg/kg), and data were collected at the indicated time points

In this study, we hypothesized that hyperoside confers neuroprotection against poly-GR-induced toxicity by restoring mitochondrial homeostasis and activating the Nrf2 antioxidant pathway. To test this hypothesis, we first examined the impact of poly-GR on mitochondrial dysfunction and redox imbalance in motor neuron-like cells. To enhance the translational significance of our findings, we further validated the neuroprotective effects of hyperoside in an established in vivo DPR-induced *C9orf72*-ALS mouse model based on AAV9-mediated EGFP-GR_50_ expression. Together, these approaches aimed to determine whether hyperoside can alleviate poly-GR-mediated neurodegeneration, providing a foundation for its development as a potential therapeutic candidate for *C9orf72*-ALS. The overall experimental design of the study is summarized in Fig. 1.

## Materials and methods

### Cell culture

The NSC-34 mouse motor neuron-like cell line, widely used for in vitro studies of ALS pathology, was obtained from CELLutions Biosystems (CLU140; Toronto, ON, Canada). Cells were cultured in Dulbecco’s modified Eagle’s medium (DMEM; Gibco, 11965-092; Thermo Fisher Scientific, Waltham, MA, USA) supplemented with 10% fetal bovine serum and 1% penicillin–streptomycin (Gibco, 15140-122). For transient transfection, NSC-34 cells were seeded in 10-cm dishes and transfected once with the plasmids listed in Table 1 using PolyJet reagent (SL100688; SignaGen Laboratories, Frederick, MD, USA) at a 2:1 reagent-to-DNA ratio. PolyJet/DNA complexes were prepared in 0.5 mL serum-free DMEM and incubated for 20 min at room temperature before being added to the cultures. NSC34 cells were transfected once and maintained for 24 or 48 h. For pharmacological intervention, cells were pretreated with hyperoside (HY-N0452; MedChemExpress, Monmouth Junction, NJ, USA) or P110 (HY-P10368; MedChemExpress, Monmouth Junction, NJ, USA), where indicated, for 3 h prior to transfection. Following transfection, cells were incubated until the 24-h endpoint and subsequently subjected to biochemical and imaging analyses, including western blotting, immunofluorescence (IF), MitoTracker, and CellROX assays. For cell viability experiments, cells underwent the same pretreatment and transfection schedule and were analyzed at 48 h post-transfection using the CCK-8 assay. An overview of the experimental workflow for both in vitro and in vivo studies is summarized in Fig. 1.

**Table 1:** Antibodies and recombinant DNA

Reagent or resource	Source	Identifier
Antibodies		
Drp1	Cell signaling	8570; RRID:AB_10950498
p-DRP1(Ser637)	Cell signaling	4867; RRID: AB_10622027
p-DRP1(Ser616)	Cell signaling	3455; RRID:AB_2085352
GAPDH	Merck	MAB374; RRID:AB_2107445
Opa1	Cell signaling	80471; RRID:AB_2734117
Nrf2	Cell signaling	12721; RRID:AB_2715528
HO-1	Cell signaling	43966; RRID:AB_2799254
GPx4	Proteintech	67763-1-Ig; RRID: AB_2909469
α-tubulin	Merck	T5168; RRID:AB_477579
C-caspase 3	Cell signaling	9661; RRID:AB_2341188
Bax	Santa cruz	sc-7480; RRID:AB_626729
Bcl-2	Santa cruz	sc-7382; RRID:AB_626736
β-actin	Merck	MAB1501; RRID:AB_2223041
TOM20	Proteintech	11802-1-AP; RRID:AB_2207530
NeuN	Merck	MAB377; RRID:AB_2298772
GFP	Rockland Immunochemicals	600–901-215; RRID:AB_1537403
Donkey anti-mouse IgG (H + L) Highly Cross-Adsorbed Secondary Antibody, Alexa Fluor^™^ Plus 555	Invitrogen	A32773; RRID: AB_2762848
Donkey anti-mouse IgG (H + L) Highly Cross-Adsorbed Secondary Antibody, Alexa Fluor^™^ Plus 488	Invitrogen	A32766; RRID: AB_2762823
Donkey anti-rabbit IgG (H + L) Highly Cross-Adsorbed Secondary Antibody, Alexa Fluor^™^ Plus 555	Invitrogen	A32794; RRID: AB_2762834
Donkey anti-rabbit IgG (H + L) Highly Cross-Adsorbed Secondary Antibody, Alexa Fluor^™^ Plus 488	Invitrogen	A32790; RRID: AB_2762833
Goat anti-mouse IgG antibody, peroxidase conjugated	Merck	AP124P; RRID:AB_90456
Goat anti-rabbit IgG antibody, peroxidase conjugated	Merck	AP132P; RRID:AB_90264
Recombinant DNA		
eGFP-GR50	This paper	N/A
pCMV3-HA	SinoBiological	CV017
pCMV3-Nrf2-HA	SinoBiological	MG56971-CY

### Animal model

To evaluate whether hyperoside exerts neuroprotective effects in vivo in the context of poly-GR-mediated neurotoxicity, we utilized a previously established DPR-induced *C9orf72*-ALS mouse model. Recombinant AAV9 carrying the hSyn-EGFP-GR_50_ construct was generated by the AAV Core Facility at Academia Sinica (Taipei, Taiwan) following established procedures. For in vivo experiments, neonatal C57BL/6J mice at postnatal day 0 (P0) received unilateral intracerebroventricular injections into the left lateral ventricle with 2 μL AAV9-hSyn-EGFP-GR₅₀ (2.1 × 10^13^ vg/mL) using a previously described protocol [5, 11]. Because viral delivery was unilateral, transgene expression and associated pathological changes were predominantly observed in the ipsilateral hemisphere. Therefore, subsequent histological analyses were performed primarily in the ipsilateral motor cortex where EGFP-GR₅₀ expression was most prominent. C57BL/6JNarl mice were obtained from the National Center for Biomodels, National Institute of Animal Resources (Taiwan), and housed in a controlled environment (22 ± 0.5 °C; 60 ± 15% humidity; 12-h light/dark cycle) with free access to food and water. All experimental procedures were approved by the Institutional Animal Care and Use Committee of China Medical University (CMUIACUC-2025-007) and conducted in accordance with institutional and international guidelines for animal care. Beginning on day in vivo 16 (DIV16), hyperoside was administered intraperitoneally at a dose of 10 mg/kg, three times per week, for a total of five injections. Mice were monitored daily and followed until humane endpoint criteria were met. Animal numbers and exclusion criteria: Each experimental group initially consisted of six mice. During brain tissue processing and cryosectioning, EGFP-GR_50_-expressing tissues exhibited increased fragility consistent with neurodegenerative pathology. Consequently, some samples were excluded from quantitative analysis due to mechanical disruption resulting in incomplete motor cortex structures. Only sections with intact and anatomically comparable motor cortex regions were included for NeuN-positive neuronal quantification. The final numbers analyzed are indicated in the corresponding figure legends.

### Western blot

Cells were collected and lysed in IP buffer (50 mM NaCl, 0.5% NP-40, 10 mM Tris–HCl, pH 8.0) supplemented with protease inhibitors, followed by incubation on ice for 30 min. Lysates were quantified to ensure equal protein loading, and samples were denatured at 95 °C for 10 min before separation by SDS–polyacrylamide gel electrophoresis using gels of appropriate acrylamide concentrations. Proteins were transferred onto polyvinylidene difluoride (PVDF) membranes and blocked with 5% Blotting-Grade Blocker (#1706404; Bio-Rad Laboratories, Hercules, CA, USA) for 1 h at room temperature. Membranes were incubated overnight at 4 °C with the primary antibodies listed in Table 1, washed with TBST, and then incubated with HRP-conjugated secondary antibodies for 1 h at room temperature. Chemiluminescent signals were detected using an Azure 400 imaging system (Azure Biosystems, Dublin, CA, USA), and band intensities were quantified using Image Studio Lite software (LI-COR, Lincoln, NE, USA) or Fiji ImageJ (version 1.53t; NIH, Bethesda, MD, USA).

### MitoTracker assay

Mitochondrial labeling was performed using MitoTracker^®^ Red CMXRos (M7512; Thermo Fisher Scientific) according to the manufacturer’s protocol. NSC-34 cells expressing EGFP or EGFP-GR_50_ were seeded in 6-cm dishes and exposed to Hyperoside or P110 as indicated. Cells were then incubated with 500 nM MitoTracker for 30 min at 37 °C under standard culture conditions, followed by three washes with PBS. Fluorescent images were acquired using an LSM 900 confocal microscope (Carl Zeiss, Oberkochen, Germany) equipped with a Plan-Apochromat 20 × objective. Identical laser power and detector settings were applied for all groups to maintain consistency. Quantification of mitochondrial fluorescence intensity was carried out using Fiji/ImageJ (version 1.53t; NIH, Bethesda, MD, USA).

### CellRox assay

Intracellular ROS levels were quantified using the CellROX oxidative stress reagent (C10422; Thermo Fisher Scientific) following the manufacturer’s instructions. NSC-34 cells expressing EGFP or EGFP-GR_50_ were seeded in 6-cm dishes and treated with Hyperoside as indicated. Cells were then incubated with 5 μM CellROX for 30 min at 37 °C under standard culture conditions and subsequently washed three times with PBS. Fluorescence signals were captured on an LSM 900 confocal microscope (Carl Zeiss, Oberkochen, Germany) equipped with a Plan-Apochromat 20 × objective. All images were acquired using identical laser and detector settings to ensure comparability across samples. Fluorescence intensity measurements were performed using Fiji/ImageJ (version 1.53t; NIH, Bethesda, MD, USA).

### Immunofluorescence analysis

NSC-34 cells were seeded onto poly-L-lysine–coated coverslips and allowed to adhere for 16 h before transfection with EGFP or EGFP-GR_50_ plasmids, with or without hyperoside or P110 treatment, as described in the cell culture section. After the designated expression or treatment periods, cells were fixed with 4% paraformaldehyde. For tissue sections, OCT-embedded samples were thawed to remove excess compound, followed by antigen retrieval at 80 °C for 10 min and permeabilization with PBS containing 0.1% Triton X-100. To minimize non-specific binding, cells and tissue sections were blocked with 10% donkey serum in PBS for 1 h at room temperature. Samples were then incubated overnight at 4 °C with the primary antibodies listed in Table 1, washed with PBS containing 0.1% Triton X-100, and subsequently incubated with fluorophore-conjugated secondary antibodies for 1 h at room temperature. Nuclei were counterstained with DAPI before the final washes and mounting. Confocal images were acquired using an LSM 900 microscope (Carl Zeiss, Oberkochen, Germany) equipped with a standard confocal module or Airyscan detector and a Plan-Apochromat 63 ×/1.3 NA objective. All images were obtained at 23 °C using the manufacturer’s acquisition software. Fluorescence intensities were quantified using Fiji/ImageJ (version 1.53t; National Institutes of Health, Bethesda, MD, USA).

### CCK8 assay

NSC-34 cells expressing EGFP, EGFP-GR_50_, or treated with 100 μM H₂O₂ or Hyperoside were maintained at 37 °C in a 5% CO₂ incubator for the indicated periods. Cell viability was assessed using the Cell Counting Kit-8 (CCK-8; ab228554, Abcam). Diluted CCK-8 reagent was added to each well and incubated for 1 h at 37 °C, after which absorbance at 450 nm was measured using a SpectraMax iD3 microplate reader (Molecular Devices).

### Molecular docking

The crystal structures of Nrf2 (PDB ID: 2FLU), Drp1 (PDB ID: 4BEJ), and Opa1 (PDB ID: 8EFS) were obtained from the Protein Data Bank and UniProt database. Protein structures were preprocessed using Discovery Studio Visualizer to remove water molecules and co-crystallized ligands prior to docking. The three-dimensional structure of hyperoside was retrieved from the PubChem database and subsequently refined using PyMOL to obtain an energetically favorable conformation. Prior to docking, the structures of Nrf2, Drp1, and hyperoside were prepared and optimized to ensure the presence of appropriate active conformations. Molecular docking analysis was then performed using AutoDock Vina version 1.2.0.

### Statistical analysis

Statistical analyses were performed using GraphPad Prism version 10.6.1 (GraphPad Software, La Jolla, CA, USA). For cell-based assays, data were collected from at least three independent biological replicates. Each group initially consisted of six mice. Final numbers used for analysis are reported in the corresponding figure legends. Survival analyses were performed using the Kaplan–Meier method, and statistical significance was assessed using the log-rank (Mantel-Cox) test and Gehan-Breslow-Wilcoxon test. Comparisons between two groups were assessed using two-tailed Student’s t-tests, whereas one-way ANOVA followed by Tukey’s multiple comparisons test was used for analyses involving more than two groups. Results are expressed as mean ± SEM, and statistical significance was defined as *P < 0.05, **P < 0.01, ***P < 0.001, and ****P < 0.0001.

## Results

### Poly-GR induces excessive mitochondrial fission and triggers oxidative stress

**Fig. 2:**
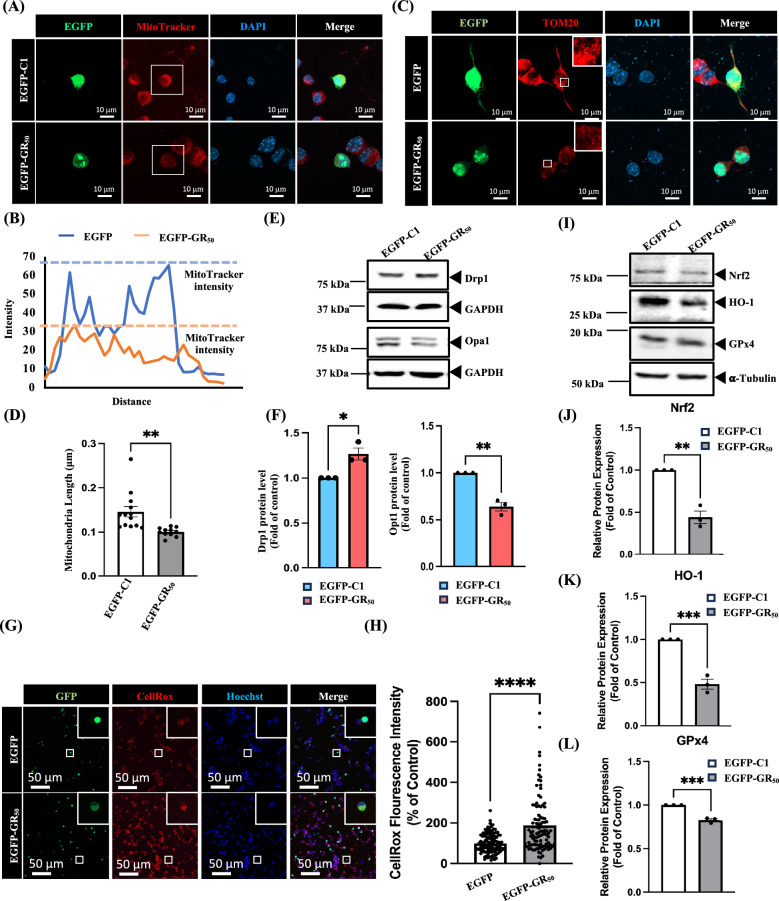
Overexpression of EGFP-GR_50_ induces mitochondrial fragmentation, oxidative stress, and dysregulation of mitochondrial homeostasis proteins in NSC34 cells. **A** Representative confocal images of NSC34 cells expressing EGFP or EGFP-GR_50_ stained with MitoTracker dye. Poly-GR_50_ markedly reduces mitochondrial membrane potential. **B** Quantification of MitoTracker fluorescence intensity indicates reduced mitochondrial membrane potential in EGFP-GR_50_-expressing cells. **C** Immunofluorescence staining of TOM20 further confirmed enhanced mitochondrial fragmentation in EGFP-GR_50_ cells. **D** Quantification of mitochondrial length shows a significant reduction following EGFP-GR_50_ expression. Data are presented as mean ± SEM and analyzed using an unpaired two-tailed t-test (**p < 0.01). N = 3 biologically independent experiments with consistent results. Total EGFP-C1 cells: 13; total EGFP-GR_50_ cells: 11. **E**, **F** Western blot analysis demonstrates increased Drp1 and decreased Opa1 protein expression in EGFP-GR_50_ cells, indicating enhanced mitochondrial fission and impaired fusion. Data are presented as mean ± SEM and analyzed using an unpaired two-tailed t-test (*p < 0.05, **p < 0.01). N = 3 biologically independent experiments with consistent results. **G** CellROX fluorescence imaging reveals elevated intracellular ROS levels in EGFP-GR_50_ cells, consistent with the induction of oxidative stress. **H** Quantification of CellROX fluorescence revealed a significant increase in ROS levels following the expression of EGFP-GR_50_. Data are presented as mean ± SEM and analyzed using an unpaired two-tailed t-test (****p < 0.0001). N = 3 biologically independent experiments with consistent results. Total EGFP-C1 cells: 120; total EGFP-GR_50_ cells: 102. **I**–**L** Western blot analysis of antioxidant proteins reveals reduced Nrf2, HO-1, and GPx4 expression in EGFP-GR_50_-expressing cells. Protein expression was normalized to α-tubulin as indicated. Data are presented as mean ± SEM and analyzed using an unpaired two-tailed t-test (**p < 0.01, ***p < 0.001). N = 3 biologically independent experiments with consistent results

Previous studies have shown that poly-GR disrupts mitochondrial structure and homeostasis [8, 22]. However, the molecular mechanisms underlying poly-GR-induced mitochondrial dysregulation and oxidative stress remain incompletely understood. In our study, EGFP-GR_50_ was overexpressed in the motor neuron-like NSC34 cell line. EGFP-GR_50_ expression was associated with a marked reduction in mitochondrial membrane potential (Fig. 2A and B) and a shortening of mitochondrial length (Fig. 2C and D). Dynamin-related protein 1 (Drp1) is a key mediator of mitochondrial fission that undergoes post-translational modifications and translocates to the outer mitochondrial membrane to facilitate fission [23]. In contrast, Opa1 is a nuclear-encoded inner-membrane GTPase essential for mitochondrial fusion, cristae maintenance, and respiratory function; pathogenic mutations in Opa1 disrupt mitochondrial integrity and cause autosomal dominant optic atrophy [24]. Consistent with these roles, Drp1 expression was upregulated, whereas Opa1 expression was reduced, in EGFP-GR_50_-expressing NSC34 cells, indicating a shift toward excessive mitochondrial fragmentation (Fig. 2E and F). EGFP-GR_50_ overexpression also increased intracellular ROS levels (Fig. 2G and H) and impaired antioxidant defenses by suppressing the Nrf2 pathway, resulting in a reduction in the expression of its downstream targets, HO-1 and GPx4 (Fig. 2I–L). Collectively, these data indicate that poly-GR disrupts mitochondrial fission and promotes oxidative stress in NSC34 cells.

### Hyperoside modulates mitochondrial fission-associated proteins in poly-GR-expressing NSC34 cells

**Fig. 3:**
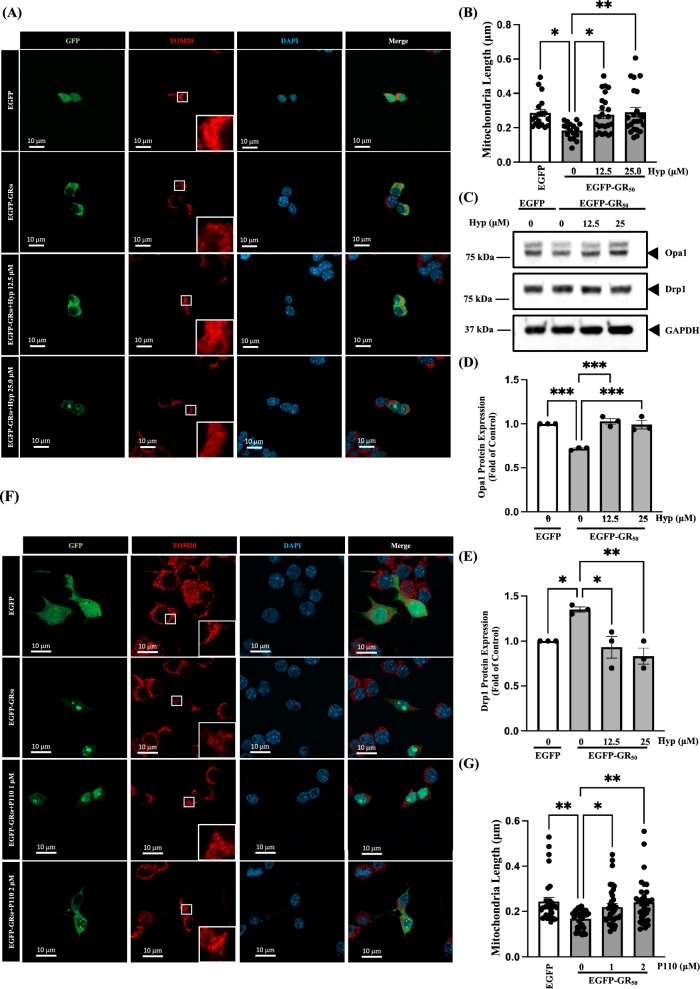
Hyperoside reverses poly-GR_50_-induced mitochondrial fragmentation by restoring the balance of Opa1 and Drp1. **A** Representative confocal images of EGFP-GR_50_-expressing cells treated with hyperoside (12.5 or 25 μM), visualized by TOM20 immunofluorescence. **B** Quantification of mitochondrial length showing hyperoside-mediated restoration of mitochondrial morphology. Data are presented as mean ± SEM and were analyzed using one-way ANOVA followed by Tukey’s multiple comparisons test (F (3, 78) = 4.423, *p* = 0.0063) (*p < 0.05, **p < 0.01). N = 3 biologically independent experiments with consistent results. Total cells analyzed: EGFP, 18; EGFP-GR_50_, 17; EGFP-GR_50_ + 12.5 μM hyperoside, 24; EGFP-GR_50_ + 25 μM hyperoside, 23. **C**–**E** Western blot analyses of Opa1 and Drp1 expression following hyperoside treatment in EGFP-GR_50_-expressing NSC34 cells. Data are presented as mean ± SEM and were analyzed using one-way ANOVA followed by Tukey’s multiple comparisons test [**D** F (3, 8) = 24.94, *p* = 0.0002]; [**E** F (3, 8) = 8.751, *p* = 0.0066] (*p < 0.05, **p < 0.01, ***p < 0.001). N = 3 biologically independent experiments with consistent results. **F** TOM20 staining of EGFP-GR_50_ cells treated with the Drp1 inhibitor P110 (1 or 2 μM). **G** Quantification of mitochondrial length after P110 treatment in EGFP-GR_50_-expressing NSC34 cells. Data are presented as mean ± SEM and were analyzed using one-way ANOVA followed by Tukey’s multiple comparisons test (F (3,131) = 6.482, *p* = 0.0004) (*p < 0.05, **p < 0.01). N = 3 biologically independent experiments with consistent results. Total cells analyzed: EGFP, 30; EGFP-GR_50_, 36; EGFP-GR_50_ + 1 μM P110, 35; EGFP-GR_50_ + 2 μM P110, 34

Hyperoside, a flavonol glycoside also known as quercetin-3-O-galactoside, is abundant in Hypericum and Crataegus species [15]. It exhibits antioxidant, anti-inflammatory, and cytoprotective activities through modulation of Nrf2, NF-κB, and mitochondrial homeostasis, supporting its therapeutic potential in oxidative stress-associated diseases [25]. However, its neuroprotective mechanisms in poly-GR-induced *C9orf72*-ALS models remain poorly defined. Prior to mechanistic investigation, we first assessed the cytotoxicity of hyperoside in NSC34 cells. Treatment with lower concentrations of hyperoside did not induce detectable cytotoxicity; however, higher concentrations (50 and 100 μM) caused a modest but statistically significant reduction in cell viability (Supplementary Fig. 1). To examine its effects on mitochondrial length, EGFP-GR_50_-expressing NSC34 cells were pretreated with hyperoside for 3 h. Hyperoside treatment restored mitochondrial length in EGFP-GR_50_-expressing NSC34 cells, as shown by TOM20 staining (Fig. 3A and B). We next assessed proteins involved in mitochondrial fission and fusion. Hyperoside significantly increased Opa1 expression and reduced total Drp1 levels, without altering Drp1 phosphorylation, in EGFP-GR_50_-expressing NSC34 cells. (Fig. 3C–E, Supplementary Fig. 4), indicating a shift toward mitochondrial fusion. In addition, treatment with P110, a selective Drp1/Fis1 interaction inhibitor and mitochondrial fission blocker, also increased mitochondrial length in poly-GR-overexpressing NSC34 cells (Fig. 3F and G), consistent with the effects of hyperoside. Collectively, these findings suggest that hyperoside reverses poly-GR-induced mitochondrial fragmentation by rebalancing the Opa1/Drp1 axis and restoring mitochondrial length.

### Hyperoside restores the Nrf2/HO-1/GPx4 antioxidant axis and rescues Nrf2 nuclear translocation impaired by poly-GR

**Fig. 4:**
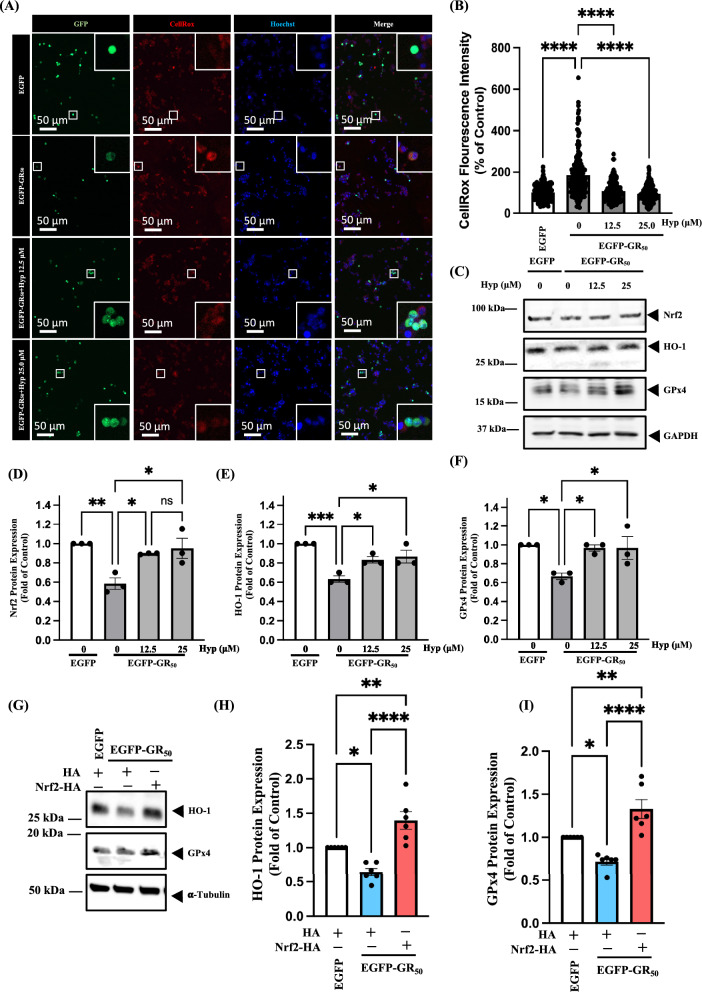
Hyperoside attenuates oxidative stress by restoring Nrf2/HO-1/GPx4 signaling in poly-GR_50_-expressing cells. **A** CellROX fluorescence images of cells treated with hyperoside in EGFP-GR_50_-expressing NSC34 cells. **B** Quantification of ROS intensity showing a significant reduction after hyperoside treatment in EGFP-GR_50_-expressing NSC34 cells. Data are presented as mean ± SEM and were analyzed using one-way ANOVA followed by Tukey’s multiple comparisons test (F (3, 619) = 69.54, *p* = 0.0001) (****p < 0.0001). N = 3 biologically independent experiments with consistent results. Total cells analyzed: EGFP, 142; EGFP-GR_50_, 195; EGFP-GR_50_ + 12.5 μM hyperoside, 141; EGFP-GR_50_ + 25 μM hyperoside, 145. **C**–**F** Western blot analyses of Nrf2, HO-1, and GPx4 protein levels following hyperoside treatment in EGFP-GR_50_-expressing NSC34 cells. Data are presented as mean ± SEM and were analyzed using one-way ANOVA followed by Tukey’s multiple comparisons test [**D** F (3, 8) = 5.867, *p* = 0.0203; **E** F (3, 8) = 13.78, *p* = 0.0016; **F** F (3, 8) = 9.627, *p* = 0.0050] (*p < 0.05, **p < 0.01, ***p < 0.001). N = 3 biologically independent experiments with consistent results. **G**–**I** Overexpression of Nrf2-HA rescues the HO-1 and GPx4 expression suppressed by EGFP-GR_50_ expression. Data are presented as mean ± SEM and were analyzed using one-way ANOVA followed by Tukey’s multiple comparisons test [**H** F (2, 15) = 21.66, *p* < 0.0001; **I** F (2, 15) = 20.41, *p* < 0.001)] (*p < 0.05, **p < 0.01, ****p < 0.0001). N = 3 biologically independent experiments with consistent results

**Fig. 5:**
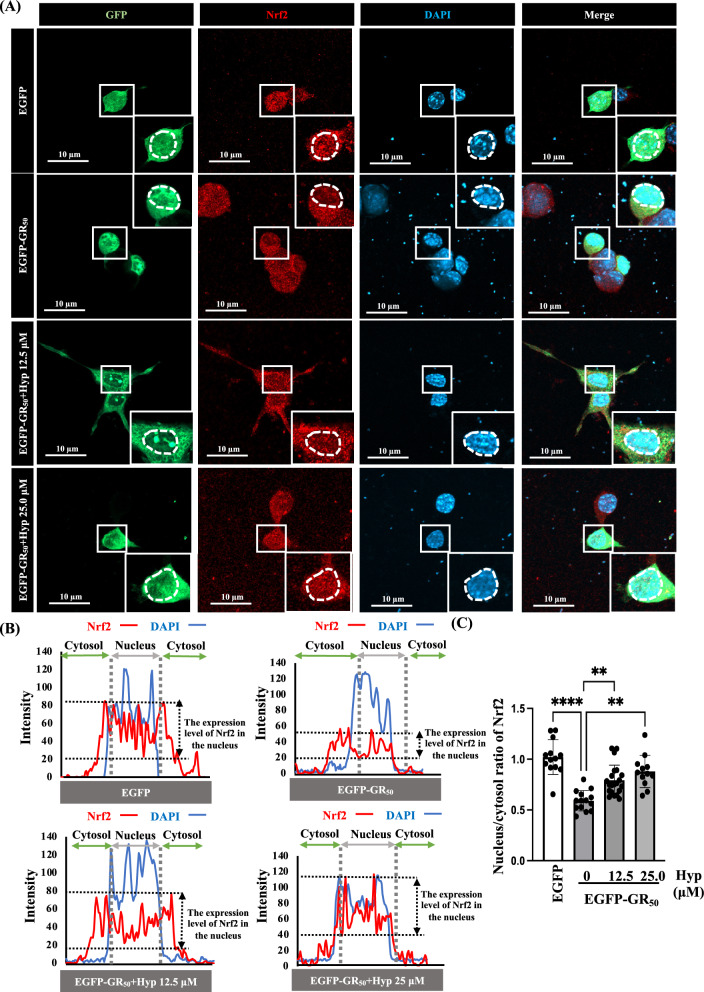
Hyperoside enhances Nrf2 nuclear translocation, which is suppressed by poly-GR_50_. **A** Representative immunofluorescence images of Nrf2 localization in EGFP- or EGFP-GR_50_-expressing cells with or without hyperoside treatment. **B** Fluorescence intensity profiles demonstrate impaired Nrf2 nuclear entry in EGFP-GR_50_ cells, rescued by hyperoside. **C** Quantification of nuclear-to-cytosolic Nrf2 ratio. Data are presented as mean ± SEM and were analyzed using one-way ANOVA followed by Tukey’s multiple comparisons test (F (3, 56) = 20.38, *p* = 0.0001) (**p < 0.01, ****p < 0.0001). N = 3 biologically independent experiments with consistent results. Total cells analyzed: EGFP, 13; EGFP-GR_50_, 14; EGFP-GR_50_ + 12.5 μM hyperoside, 21; EGFP-GR_50_ + 25 μM hyperoside, 12

Previous studies have reported that poly-GR induces oxidative stress and contributes to neuronal death [10]. However, whether hyperoside can attenuate poly-GR-induced oxidative stress remains unclear. In NSC34 cells, hyperoside treatment significantly reduced EGFP-GR_50_-induced ROS accumulation (Fig. 4A and B). We next examined key components of the antioxidant pathway, including Nrf2, GPx4, and HO-1. Hyperoside restored the expression of Nrf2, GPx4, and HO-1 in EGFP-GR_50_-expressing NSC34 cells (Fig. 4C–F). To further determine whether the reduction of HO-1 and GPx4 in EGFP-GR_50_-expressing cells occurs through impaired Nrf2 signaling, Nrf2 was overexpressed in NSC34 cells. Nrf2 overexpression rescued the poly-GR-mediated decrease in HO-1 and GPx4, confirming that poly-GR disrupts downstream antioxidant defenses by inhibiting Nrf2 (Fig. 4G–I). Poly-GR has also been shown to alter nucleocytoplasmic transport, thereby exacerbating neuronal toxicity [26, 27]. Whether poly-GR affects Nrf2 nuclear translocation and whether hyperoside can restore this process remains unknown. We found that poly-GR markedly reduced Nrf2 nuclear localization, whereas hyperoside treatment promoted Nrf2 translocation into the nucleus in EGFP-GR_50_-expressing NSC34 cells (Fig. 5A). Line-profile analysis and quantification of the nuclear-to-cytosolic ratio further confirmed increased nuclear accumulation of Nrf2 following hyperoside treatment (Fig. 5B and C). Collectively, these data indicate that poly-GR not only suppresses the Nrf2/HO-1/GPx4 antioxidant axis but also impairs Nrf2 nuclear translocation, thereby promoting oxidative stress. Hyperoside effectively counteracts these effects by enhancing Nrf2 nuclear translocation and restoring downstream antioxidant gene expression, ultimately reducing poly-GR-induced oxidative stress.

### Hyperoside reduces poly-GR-induced apoptosis by restoring mitochondrial membrane potential

**Fig. 6:**
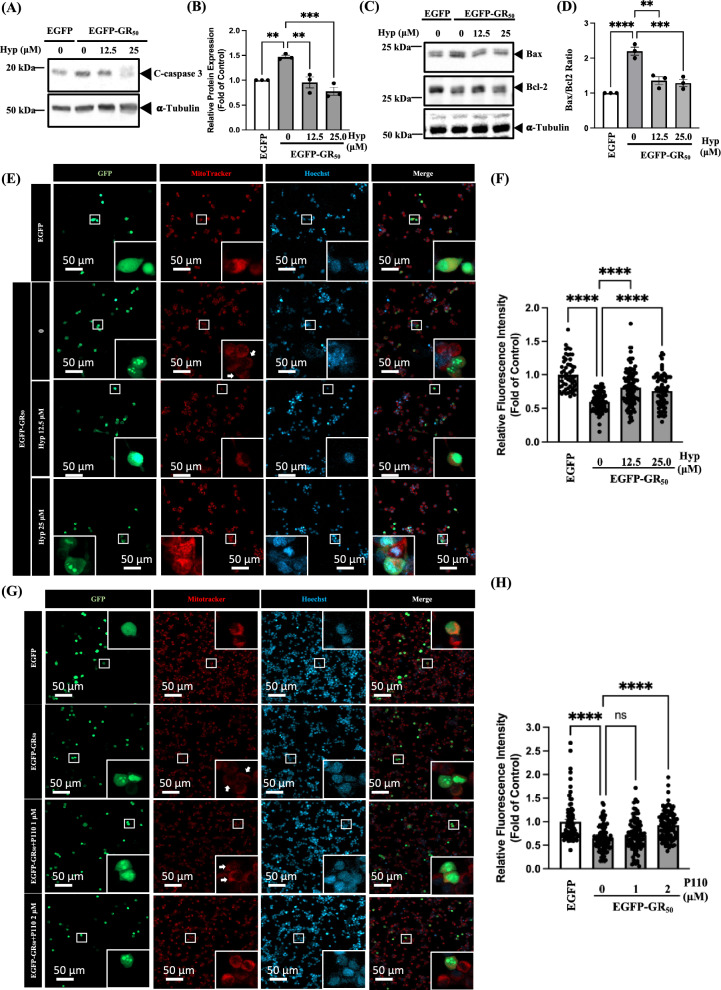
Hyperoside suppresses poly-GR_50_-induced apoptosis and preserves mitochondrial membrane potential. **A**–**D** Western blot analysis of cleaved caspase-3, Bax, and Bcl-2 protein levels, normalized to α-tubulin as the loading control. Hyperoside reduced the expression of cleaved caspase-3 and Bax, while increasing Bcl-2 levels. Data are presented as mean ± SEM and were analyzed using one-way ANOVA followed by Tukey’s multiple comparisons test [**B** F (3, 8) = 17.69, *p* = 0.0007; **D** F (3, 8) = 29.71, *p* = 0.0001] (*p < 0.05, **p < 0.01, ***p < 0.001, ****p < 0.0001). N = 3 biologically independent experiments with consistent results. **E–F** MitoTracker imaging and quantification of mitochondrial membrane potential following hyperoside treatment in EGFP-GR_50_-expressing NSC34 cells. Data are presented as mean ± SEM and were analyzed using one-way ANOVA followed by Tukey’s multiple comparisons test [**F** F (3, 329) = 35.32, *p* < 0.0001] (****p < 0.0001). N = 3 biologically independent experiments with consistent results. Total cells analyzed: EGFP, 55; EGFP-GR_50_, 83; EGFP-GR_50_ + 12.5 μM hyperoside, 108; EGFP-GR_50_ + 25 μM hyperoside, 87. **G**, **H** P110 treatment similarly restores mitochondrial membrane potential in EGFP-GR_50_-expressing NSC34 cells. Data are presented as mean ± SEM and were analyzed using one-way ANOVA followed by Tukey’s multiple comparisons test [**H** F (3, 342) = 20.87, *p* < 0.0001] (****p < 0.0001). N = 3 biologically independent experiments with consistent results. Total cells analyzed: EGFP, 77; EGFP-GR_50_, 94; EGFP-GR_50_ + 1 μM P110, 85; EGFP-GR_50_ + 2 μM P110, 90

We next investigated whether hyperoside can reduce poly-GR-induced cell death. As shown in Fig. 6A and B, cleaved caspase-3 levels were markedly elevated in EGFP-GR_50_-expressing NSC34 cells, whereas hyperoside treatment significantly reduced cleaved caspase-3 expression. In addition, the ratio of the pro-apoptotic protein Bax to the anti-apoptotic protein Bcl-2 was increased following EGFP-GR_50_ overexpression, and hyperoside treatment effectively decreased this ratio (Fig. 6C and D). Given our earlier findings that hyperoside alleviates poly-GR-induced mitochondrial fragmentation (Fig. 3), we further examined whether hyperoside can rescue mitochondrial membrane potential in EGFP-GR_50_-expressing NSC34 cells. EGFP-GR_50_ overexpression significantly reduced mitochondrial membrane potential, as determined by MitoTracker staining, whereas hyperoside treatment restored mitochondrial membrane potential (Fig. 6E and F). Consistently, treatment with P110, a selective Drp1 inhibitor, also rescued the poly-GR-induced reduction in mitochondrial membrane potential (Fig. 6G and H). Collectively, these results demonstrate that poly-GR decreases mitochondrial membrane potential, thereby promoting neuronal apoptosis, while hyperoside counteracts these effects by restoring mitochondrial membrane potential and reducing apoptotic signaling.

### Hyperoside and P110 protect poly-GR-expressing NSC34 cells from H₂O₂-induced cytotoxicity

**Fig. 7:**
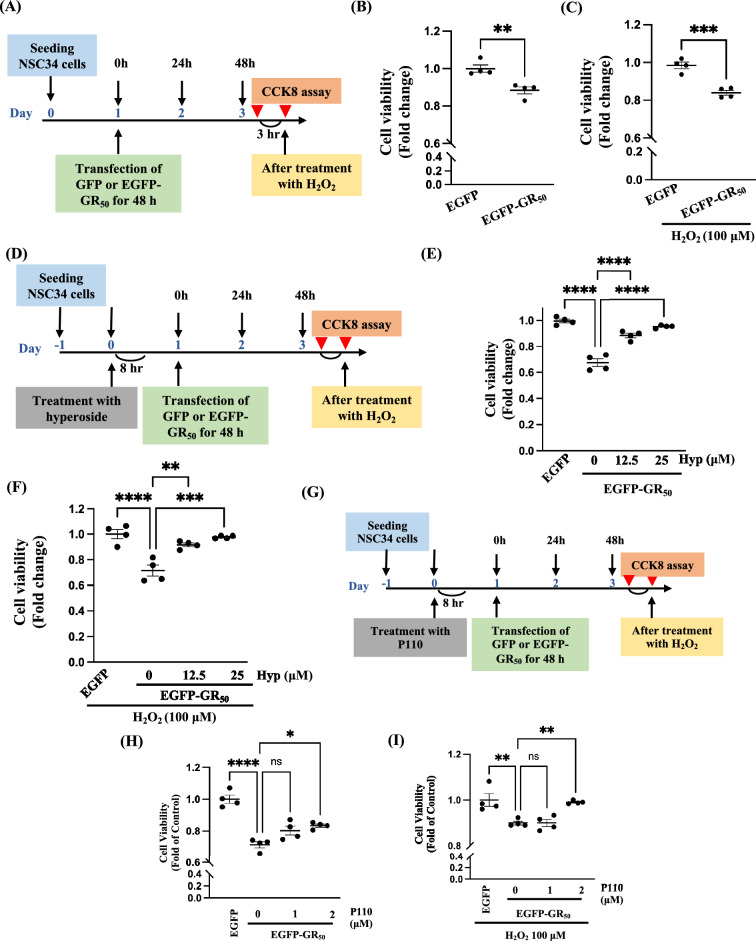
Hyperoside improves cell viability under oxidative stress in EGFP-GR_50_-expressing NSC34 cells. **A**–**C** The H₂O₂-induced oxidative challenge in EGFP-GR_50_-expressing NSC34 cells. Notably, both untreated and H₂O₂-treated EGFP-GR_50_ cells exhibit increased cell death. Data are presented as mean ± SEM and analyzed using an unpaired two-tailed t-test (**p < 0.01, ***p < 0.001). N = 4 biologically independent experiments with consistent results. **D**–**F** Hyperoside restores cell viability in EGFP-GR_50_-expressing NSC34 cells, both with and without H₂O₂ exposure. Data are presented as mean ± SEM and were analyzed using one-way ANOVA followed by Tukey’s multiple comparisons test [(**E** F (3, 12) = 58.93, *p* < 0.0001; **F** F (3, 12) = 19, 38, p < 0.0001) (**p < 0.01, ***p < 0.001, ****p < 0.0001). N = 4 biologically independent experiments with consistent results. **G**–**I** Drp1 inhibition by P110 also improves viability under oxidative stress in EGFP-GR_50_-expressing NSC34 cells. Data are presented as mean ± SEM and were analyzed using one-way ANOVA followed by Tukey’s multiple comparisons test [**H** F (3, 12) = 29.62, *p* < 0.0001; **I** F (3, 12) = 11.35, *p* = 0.0008] (*p < 0.05, **p < 0.01, ****p < 0.0001). N = 4 biologically independent experiments with consistent results

Oxidative stress is known to contribute to motor neuron degeneration in *C9orf72*-ALS [5, 11, 28]; therefore, H₂O₂ was used to mimic an oxidative stress environment. EGFP-GR_50_ overexpression significantly reduced NSC34 cell viability under both basal and H₂O₂-treated conditions (Fig. 7A, B). Hyperoside treatment effectively protected against poly-GR-induced cytotoxicity, with or without H₂O₂ exposure (Fig. 7C, D). Similarly, P110, a selective Drp1 inhibitor, also improved cell survival in EGFP-GR_50_-expressing NSC34 cells under both conditions (Fig. 7E, F). Collectively, these findings suggest that hyperoside enhances the survival of motor neuron-like NSC34 cells by mitigating oxidative stress and restoring mitochondrial fusion in the context of poly-GR overexpression.

### Hyperoside reduces neurodegeneration in poly-GR-induced *C9orf72*-ALS

**Fig. 8:**
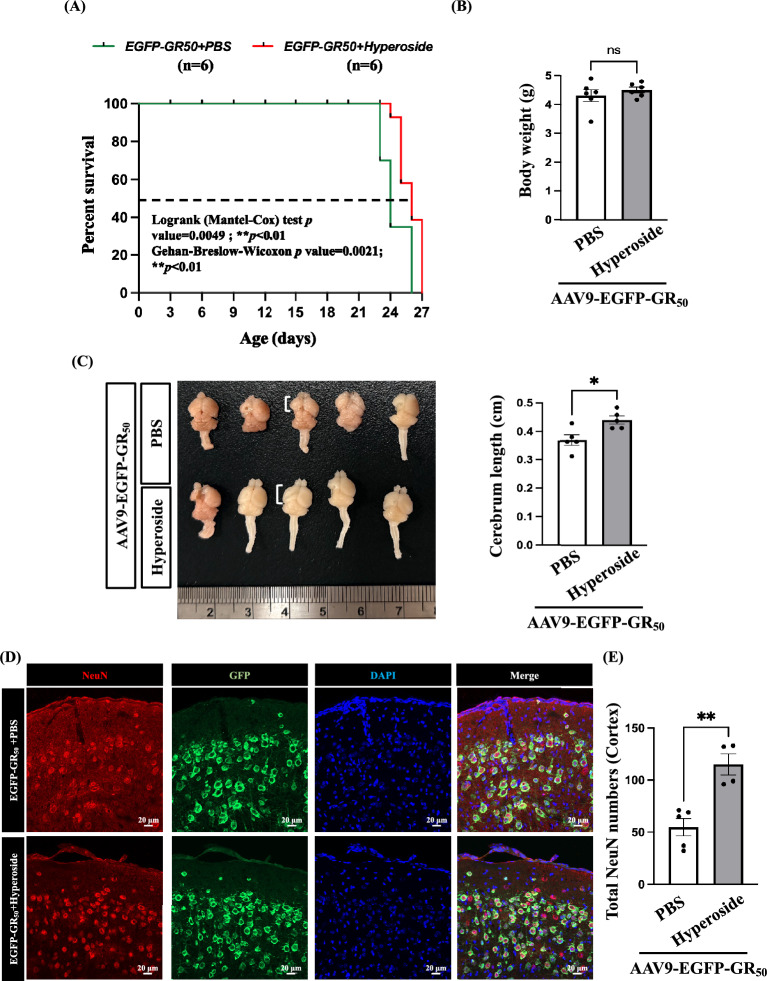
Hyperoside modestly improves survival and increases neuronal numbers in the AAV9-EGFP-GR_50_ mouse model of *C9orf72*-ALS. **A** Kaplan–Meier survival curves show a significant survival benefit in hyperoside-treated EGFP-GR_50_ mice. Statistical analyses were performed using log-rank (Mantel-Cox) and Gehan-Breslow-Wilcoxon tests (n = 6 per group). **B** Quantification of body weight in PBS-treated EGFP-GR_50_ mice (n = 6) and hyperoside-treated EGFP-GR_50_ mice (n = 6). **C** Representative images of brain structures from PBS- or hyperoside-treated EGFP-GR_50_ mice. PBS-treated EGFP-GR_50_ mice exhibited a shorter left cerebral hemisphere length compared with the hyperoside-treated group. Quantification of left cerebrum length in PBS- and hyperoside-treated EGFP-GR_50_ mice (n = 5). Data are presented as mean ± SEM and analyzed using an unpaired two-tailed t-test (*p < 0.05). **D**, **E** Representative immunofluorescence images showing NeuN-positive neurons in the motor cortex of PBS- or hyperoside-treated EGFP-GR_50_ mice. Quantification of NeuN-positive neuronal numbers in the motor cortex of PBS-treated (n = 5) and hyperoside-treated (n = 4) EGFP-GR_50_ mice. Data are presented as mean ± SEM and analyzed using an unpaired two-tailed t-test (**p < 0.01)

Based on our in vitro findings, hyperoside alleviates mitochondrial dysfunction, reduces oxidative stress, and protects neuronal survival. To further evaluate its neuroprotective effects in vivo, we employed an AAV-based system to express EGFP-GR_50_ via intracerebroventricular (ICV) injection in P0 mouse pups. Hyperoside treatment modestly extended overall survival, reducing the 50% mortality rate by 7.4% in the poly-GR-induced *C9orf72*-ALS mouse model (Fig. 8A). Although no significant differences in body weight were observed between groups (Fig. 8B), hyperoside-treated mice showed a modest increase in left cerebral hemisphere length and better-preserved structural integrity, indicative of attenuated brain atrophy (Fig. 8C). Histological analyses further revealed a significant increase in neuronal numbers in hyperoside-treated EGFP-GR_50_ mice compared with untreated EGFP-GR_50_ controls (Fig. 8D and E). Collectively, these data indicate that hyperoside mitigates neurodegeneration in vivo, supporting its therapeutic potential and functional protection in the poly-GR-induced *C9orf72*-ALS model.

### Hyperoside exhibits potential binding interactions with Nrf2 and Drp1

Consistent with our experimental findings, hyperoside increased Nrf2 expression and promoted its nuclear translocation, while also modulating Drp1 expression in EGFP-GR_50_-expressing NSC34 cells (Figs. 3C, 4C, and 5). To further explore the potential molecular basis of these effects, in silico molecular docking analyses were performed. As shown in Supplementary Fig. 2, hyperoside displayed favorable predicted binding affinities toward Nrf2 (−8.8 kcal/mol) and Drp1 (−7.1 kcal/mol), whereas a lower binding affinity was observed for Opa1 (−5.8 kcal/mol; Supplementary Fig. 3). Docking models suggested that hyperoside can be accommodated within putative binding regions of Nrf2 and Drp1 through non-covalent interactions. Although these in silico data do not demonstrate direct biochemical binding, they provide structural support for the potential involvement of hyperoside in modulating Nrf2- and Drp1-associated signaling pathways.

## Discussion

In this study, we demonstrate that the flavonoid hyperoside attenuates poly-GR-induced mitochondrial fragmentation, oxidative stress, and neurotoxicity, highlighting the holistic, multi-target pharmacological profile typical of phytochemicals and underscoring their therapeutic potential in neurodegenerative disorders, particularly *C9orf72*-associated ALS. Our data demonstrate that poly-GR markedly downregulates Opa1 while upregulating Drp1, thereby promoting excessive mitochondrial fission. This imbalance results in mitochondrial fragmentation and impaired mitochondrial functional integrity. Under physiological conditions, mitochondrial fission serves as a quality control mechanism that facilitates the segregation and removal of damaged mitochondria. However, dysregulated or excessive fission may overwhelm these protective mechanisms, leading to mitochondrial dysfunction and increased oxidative stress [29, 30]. These findings are consistent with the mitochondrial morphological alterations and quantitative analyses presented in Figs. 2 and 3. In parallel, poly-GR impairs the Nrf2/HO-1/GPx4 antioxidant axis and significantly suppresses Nrf2 nuclear translocation, resulting in pronounced ROS accumulation (Figs. 4 and 5). Hyperoside effectively counteracted these pathological effects by restoring mitochondrial morphology, improving membrane potential, and reducing ROS production, thereby rescuing both mitochondrial and redox homeostasis. This dual regulation suggests that hyperoside may act upstream of both the mitochondrial fission machinery and antioxidant signaling pathways, positioning it as a promising modulator of multiple toxic cascades triggered by arginine-rich DPRs. In addition to these mitochondrial and redox benefits, hyperoside also attenuated apoptotic signaling, as evidenced by normalization of the Bax/Bcl-2 ratio and reduced cleaved caspase-3 (Fig. 6), further supporting its neuroprotective role (Fig. 7). In vivo, hyperoside treatment extended survival and increased neuronal numbers in the motor cortex of poly-GR mice (Fig. 8), indicating that its molecular actions translate into meaningful neuroprotection. Collectively, our findings demonstrate that hyperoside mitigates poly-GR toxicity by concurrently modulating mitochondrial homeostasis, oxidative defense, and apoptotic pathways, three major cellular processes disrupted in *C9orf72*-ALS. This multi-layered protection provides a strong rationale for further investigation of hyperoside as a candidate therapeutic compound targeting DPR-induced *C9orf72*-ALS pathology.

Our findings demonstrate that hyperoside mitigates mitochondrial fission, oxidative stress, and neurotoxicity in poly-GR-expressing NSC34 cells. However, whether hyperoside can also modulate other brain-resident cell types, such as astrocytes and microglia, which play central roles in neuroinflammation [11, 31, 32], remains to be clarified. Previous studies have reported that hyperoside reduces β-amyloid-induced astrocyte activation in APP/PSEN1 mice [19, 33] and attenuates astrocytic reactivity in neuropathic pain models [34]. In addition, hyperoside alleviates macrophage- and microglia-mediated neuroinflammation and oxidative stress after spinal cord injury [35] and suppresses DNA-induced microglial activation in photoreceptor degeneration [36]. Together, these observations suggest that hyperoside not only protects neuronal cells but also dampens glial activation, thereby reducing neuroinflammatory signaling. Based on this evidence, future studies should investigate whether hyperoside can similarly suppress astrocyte and microglial activation in poly-GR-driven *C9orf72*-ALS pathology.

The present study showed that poly-GR induced a shift toward mitochondrial fission in NSC34 cells, as evidenced by shortened mitochondrial length, increased Drp1 expression, and decreased Opa1 expression. In addition, poly-GR markedly reduced the protein expression of Nrf2. Previous studies have demonstrated that Nrf2 is a key regulator of mitochondrial function and cellular redox homeostasis [37]. Nrf2 promotes the expression of antioxidant enzymes, including superoxide dismutase (SOD), catalase, and glutathione peroxidase (GPx) [38], thereby limiting mitochondrial oxidative stress and maintaining mitochondrial membrane potential. Moreover, Nrf2 has been reported to regulate mitochondrial biogenesis through transcriptional control of peroxisome proliferator-activated receptor gamma coactivator-1 alpha (PGC-1α), as well as nuclear respiratory factor 1 (NRF1) and mitochondrial transcription factor A (TFAM), which are essential for mitochondrial DNA replication and transcription. Through these mechanisms, Nrf2 contributes to the maintenance of mitochondrial integrity and function. Consistent with these findings, our results suggest that poly-GR-induced downregulation of Nrf2 may impair mitochondrial homeostasis and contribute to mitochondrial dysfunction in NSC34 cells [39].

Our results demonstrate that hyperoside modulates the expression of key regulators of mitochondrial morphology, including Drp1 and Opa1, and partially rescues mitochondrial fragmentation induced by poly-GR. However, it is important to note that Drp1 activity is primarily regulated by post-translational modifications, such as phosphorylation at Ser616 and Ser637 [40, 41], rather than total protein levels alone. In the present study, hyperoside did not significantly alter Drp1 phosphorylation at these sites, suggesting that its effects are not mediated through direct regulation of Drp1 activation (Supplementary Fig. 4). Therefore, our findings primarily support an effect on mitochondrial fission-associated processes and protein expression, rather than a global restoration of mitochondrial homeostasis. Hyperoside may instead influence mitochondrial morphology through modulation of protein abundance or through alternative regulatory pathways.

Currently, Riluzole and Edaravone remain the only FDA-approved treatments for ALS [42–44]. Riluzole primarily acts as a glutamate release inhibitor and glutamate receptor antagonist [45], whereas Edaravone functions as a potent free radical scavenger targeting redox imbalance [46]. Compared with these agents, our findings indicate that hyperoside exerts broader neuroprotective actions by simultaneously reducing oxidative stress, restoring mitochondrial homeostasis, and attenuating poly-GR-induced neurotoxicity. Beyond our study, hyperoside has been reported to suppress ferroptosis triggered by zearalenone [47] and to enhance autophagic activity [48], further supporting its diverse cytoprotective properties. These observations suggest that hyperoside acts as a multi-layer phytochemical agent capable of modulating several ALS-relevant pathogenic mechanisms, including oxidative stress, mitochondrial dysfunction, ferroptosis, and autophagy impairment [8, 11, 28, 49]. Such pleiotropic actions may offer a therapeutic advantage over single-target drugs. Importantly, because hyperoside and Riluzole intervene in complementary pathways, hyperoside targets mitochondrial and redox homeostasis, and Riluzole modulates glutamatergic excitotoxicity; a combination therapy may provide enhanced benefits, particularly for *C9orf72*-associated ALS.

Previous pharmacokinetic analyses have shown that intranasal (IN) administration markedly enhances the delivery of hyperoside to the brain [19]. In contrast, intravenous (IV) injection at 20 mg/kg resulted in a brain C_max_ of 12.82 ± 0.40 ng/g, with a T_max_ of 2 h and a t_1/2_ of 10.48 ± 1.63 h [19]. Although both routes successfully achieved brain exposure, IN administration elicited more robust pharmacodynamic effects, including reduced Aβ accumulation and attenuated astrocytic activation in the cortex and hippocampus of APP/PSEN1 mice [19]. These findings suggest that IN delivery may offer a more efficient strategy for enhancing hyperoside bioavailability and central therapeutic action, particularly for neurodegenerative disorders such as *C9orf72*-associated ALS.

In this study, we generated an AAV-mediated mouse model expressing poly-GR (EGFP-GR_50_) under the control of the human synapsin promoter, enabling neuron-specific expression in the central nervous system. This model allowed us to evaluate the neuroprotective effects of hyperoside against poly-GR-induced neuronal toxicity in vivo. Our results further demonstrate that hyperoside provides measurable neuroprotection in this aggressive poly-GR_50_ ICV model. Although the extension of survival was modest (Fig. 8A), the improvement remained statistically significant, indicating that hyperoside mitigates poly-GR-induced neurotoxicity, likely through reducing mitochondrial fragmentation and oxidative stress. However, the severe toxicity caused by poly-GR resulted in early mortality, which limited the feasibility of performing behavioral assessments such as grid and hanging wire tests to evaluate motor function. Despite this limitation, the observed survival benefit highlights the biological relevance of hyperoside and provides important in vivo evidence supporting its protective effects against poly-GR-induced neuronal degeneration.

Beyond administration routes, exosome-based delivery systems represent an additional promising platform for enhancing CNS delivery of phytochemicals. Exosomes exhibit high drug-loading capacity, excellent biocompatibility, and an intrinsic ability to cross the blood–brain barrier [50]. Their natural capacity to encapsulate diverse cargos, including phytochemicals, nucleotides, and amino acids, further supports their suitability for transporting bioactive compounds such as hyperoside [51–53]. Advances in exosome engineering now enable surface modifications to enhance cell-type specificity, thereby improving neuronal targeting while minimizing off-target distribution and adverse effects. However, motor neuron selective surface markers remain insufficiently defined. Identifying such markers will be essential for the development of next-generation exosome-based delivery strategies tailored for *C9orf72*-ALS and other motor neuron disorders.

In addition to neuronal pathology, we evaluated potential glial responses in the AAV9-hSyn-EGFP-GR_50_ model by examining astrocytic (GFAP) and microglial (Iba1) markers. Interestingly, under conditions of EGFP-GR_50_ expression, neither PBS nor hyperoside treatment significantly altered GFAP-positive astrocyte abundance or Iba1 immunoreactivity (Supplementary Fig. 5). These findings suggest that, in this model, the predominant pathological changes are neuron-centered rather than driven by overt gliosis. Although hyperoside has been reported to exert anti-inflammatory effects in other experimental systems [33, 35, 54], the current model is characterized by robust, neuron-specific poly-GR expression under the hSyn promoter, which primarily induces intrinsic neuronal stress and degeneration. In such a severe and neuron-targeted paradigm, glial activation may represent a secondary or delayed response that is less susceptible to pharmacological modulation within the examined time frame. Therefore, our data support the conclusion that the neuroprotective effects of hyperoside observed in this study are largely mediated through neuron-autonomous mechanisms. Future studies employing models with more pronounced neuroinflammatory components or longer disease progression may further clarify the potential impact of hyperoside on glial-mediated inflammation.

Despite proposing a promising therapeutic strategy for poly-GR-induced *C9orf72*-ALS, several limitations should be acknowledged. First, this study primarily focused on the pathogenic mechanisms driven by poly-GR, one of the most toxic dipeptide repeat proteins associated with C9orf72 expansion. Although NSC34 cells are widely used as a motor neuron-like model in ALS research, they do not fully recapitulate the physiological complexity of primary motor neurons or patient-derived iPSC models. Patient-derived iPSC motor neurons represent a more physiologically relevant disease model, encompassing key pathological features such as RNA foci, haploinsufficiency, and multiple DPR species; however, their limited accessibility and technical challenges currently restrict their widespread use. Moreover, such complex systems are less suitable for dissecting the isolated contribution of specific DPR species, such as poly-GR. Therefore, the controlled overexpression system used in this study provides a practical and reductionist approach that enables precise mechanistic analysis of poly-GR toxicity. Future studies using primary motor neurons or *C9orf72*-ALS patient-derived iPSC models will be valuable to further validate the neuroprotective effects of hyperoside. In addition, the in vivo poly-GR model used in this study exhibits a rapidly progressive and severe phenotype. Although hyperoside treatment improved neuronal survival and modestly extended lifespan, the short survival window limited the feasibility of comprehensive behavioral assessments. Therefore, further studies using models with slower disease progression will be necessary to determine whether hyperoside can also improve motor function and long-term neurological outcomes. To further deepen the mechanistic understanding of hyperoside-mediated neuroprotection, future studies may incorporate advanced methodologies. For example, high-resolution live-cell imaging could provide dynamic insights into mitochondrial fission events and morphological changes in real time. In addition, comprehensive bioenergetic profiling, such as Seahorse-based metabolic flux analysis, would allow more precise evaluation of mitochondrial respiratory function and energy metabolism. In parallel, hyperoside is a multifunctional phytochemical with pleiotropic biological activities. While our results identify key antioxidant and mitochondrial pathways modulated by hyperoside, it is possible that additional downstream targets or compensatory mechanisms also contribute to its neuroprotective effects. Therefore, integrative approaches such as transcriptomic and proteomic profiling will be essential to delineate these broader molecular networks and fully elucidate the multi-target mechanisms underlying hyperoside action. Together, these strategies will provide a more comprehensive understanding of hyperoside-mediated neuroprotection and further support its potential as a therapeutic candidate. Ultimately, the present study primarily focused on neuronal vulnerability. Given the emerging roles of astrocytes, microglia, and oligodendrocytes in *C9orf72*-ALS pathology, a limitation of this study is that glial responses were not extensively characterized beyond GFAP and Iba1 analyses. Although no significant changes were observed in this neuron-targeted model, future studies using milder or more progressive paradigms are needed to determine whether hyperoside also modulates poly-GR-induced stress responses in glial cells. Addressing these limitations will provide a more complete understanding of hyperoside’s therapeutic potential in *C9orf72*-associated ALS.

## Conclusions

Our findings demonstrate that poly-GR expression induces mitochondrial fission, oxidative stress, and neurodegeneration. Poly-GR increases Drp1 and suppresses Opa1, resulting in excessive mitochondrial fragmentation and loss of membrane potential. In parallel, poly-GR disrupts the Nrf2/HO-1/GPx4 antioxidant axis, impairing Nrf2 nuclear translocation and thereby weakening the cellular defense machinery. Together, these convergent pathways promote neuronal cell death. Importantly, hyperoside treatment mitigates mitochondrial fragmentation and oxidative stress, ultimately protecting against poly-GR-induced neurotoxicity. These results suggest that hyperoside may serve as a potential therapeutic strategy for targeting poly-GR-driven pathogenic mechanisms in *C9orf72*-associated ALS.

## Supplementary Information

Supplementary material 1.

Supplementary material 2.

## Data Availability

Data supporting the findings of this study are available from the corresponding author upon reasonable request.

## Abbreviations

C9orf72: Chromosome 9 open reading frame 72
RAN: Repeat-associated non-AUG
DPRs: Dipeptide repeat proteins
CCK-8: Cell Counting Kit-8
ALS: Amyotrophic lateral sclerosis
Poly-GR: Poly-glycine-arginine
ROS: Reactive oxygen species
H2O2: Hydrogen peroxide
AAV: Adeno-associated virus
ICV: Intracerebroventricular
IN: Intranasal
iPSC: Induced pluripotent stem cell
EGFP: Enhanced green fluorescent protein
HA: Hemagglutinin tag
Nrf2: Nuclear factor erythroid 2-related factor 2
HO-1: Heme oxygenase 1
GPx4: Glutathione peroxidase 4
Drp1: Dynamin-related protein 1
Opa1: OPA1 mitochondrial dynamin like GTPase
PVDF: Polyvinylidene difluoride
SOD: Superoxide dismutase
GPx: Glutathione peroxidase
PGC-1α: Peroxisome proliferator-activated receptor gamma coactivator-1 alpha
NRF1: Nuclear respiratory factor 1
TFAM: Mitochondrial transcription factor A

## Declarations

### Generative AI and AI-assisted technologies

During the preparation of this manuscript, the authors used ChatGPT for language editing. All AI-assisted outputs were carefully reviewed, verified, and revised by the authors, who take full responsibility for the final content of the publication.
