# Supplementary material for: Hyperoside protects against poly-GR-mediated neurodegeneration via regulation of mitochondrial fission and oxidative stress in C9orf72-associated ALS
Source: Chin Med. 2026 Jun 4;21:161. doi: 10.1186/s13020-026-01433-w (PMC13235191; doi:10.1186/s13020-026-01433-w)
Supplement: Supplementary file 1 — Supplementary material 1. [file 13020_2026_1433_MOESM1_ESM.docx]

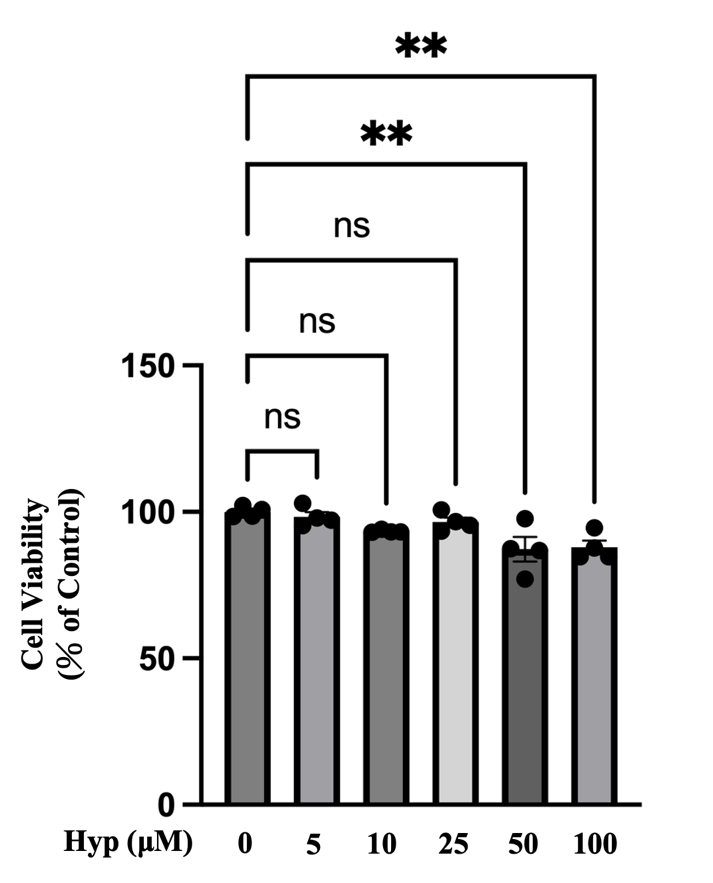


**Supplementary Figure 1. Effect of hyperoside on cytotoxicity in NSC34 cells.** Dose-response analysis of hyperoside on NSC34 cell viability. Cell viability was assessed using the CCK-8 assay. Data are presented as mean ± SEM and were analyzed by one-way ANOVA followed by Tukey’s multiple comparisons test (***p* < 0.01).


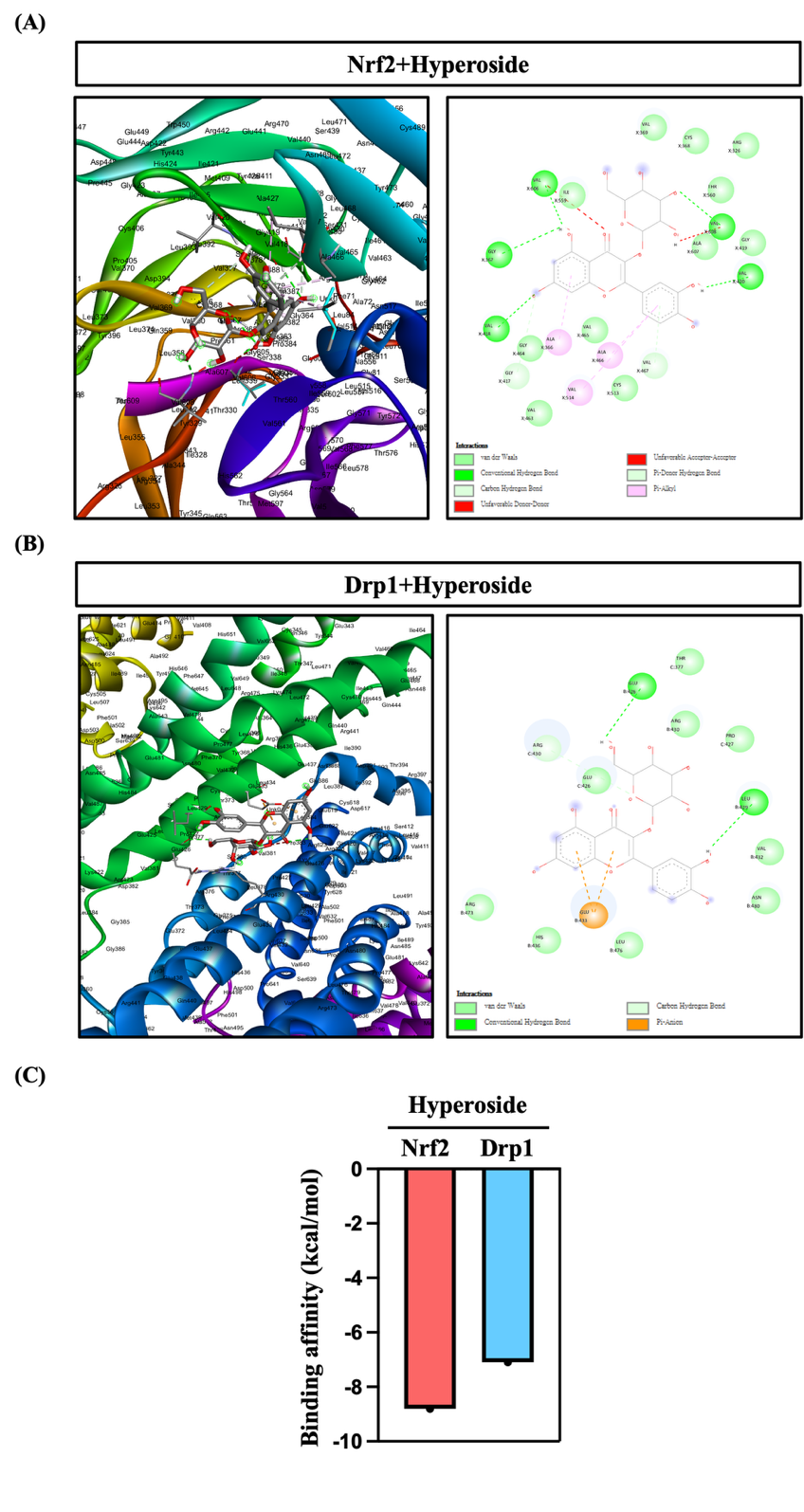


**Supplementary Figure 2. In silico molecular docking analysis of hyperoside with Nrf2 and Drp1.** (A) Predicted binding pose of hyperoside within the Nrf2 protein (PDB ID: 2FLU). (B) Predicted binding pose of hyperoside within the Drp1 protein (PDB ID: 4BEJ). (C) Predicted binding affinities of hyperoside toward Nrf2 and Drp1 as determined by AutoDock Vina. Protein-ligand interactions and binding conformations were visualized using Discovery Studio Visualizer. Docking analysis was performed to provide structural support for the potential interaction between hyperoside and Nrf2- and Drp1-associated pathways.


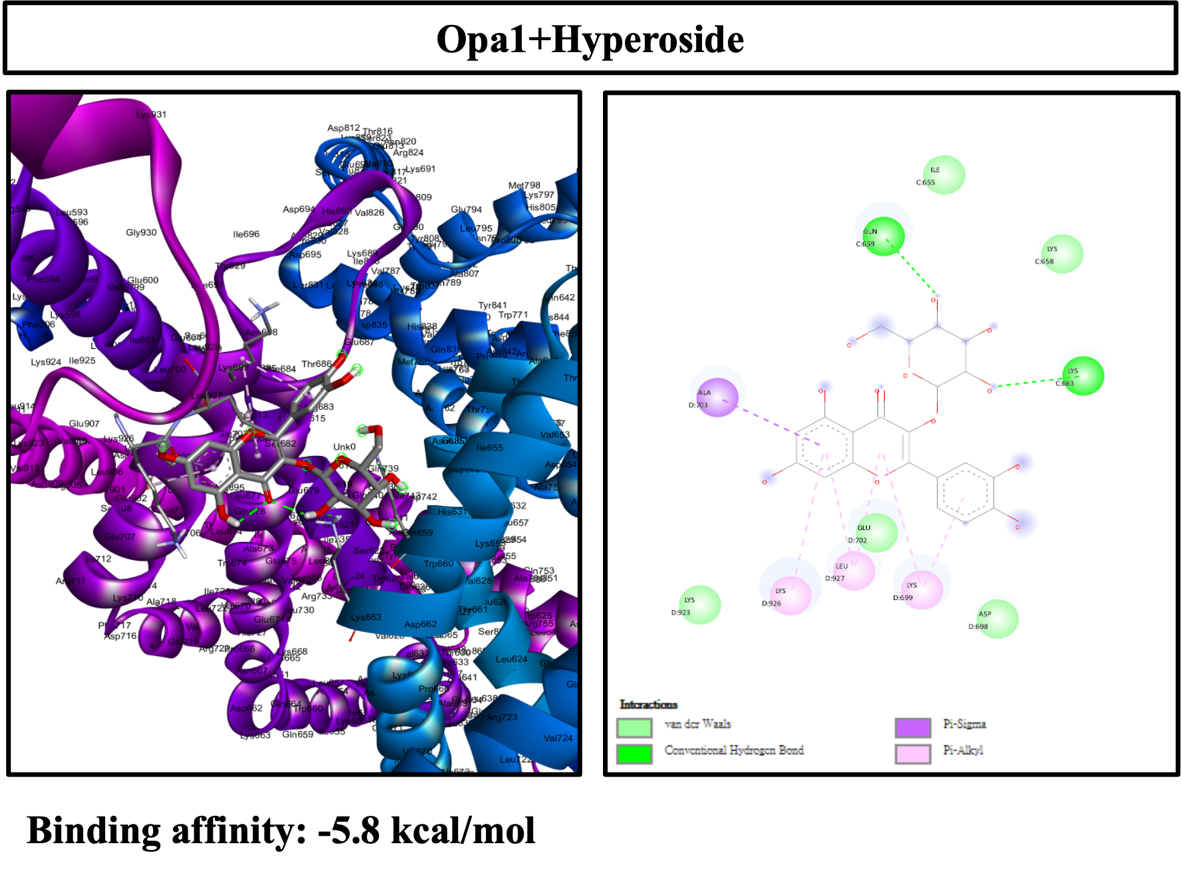


**Supplementary Figure 3. In silico molecular docking analysis of hyperoside with Opa1.** Predicted binding of hyperoside to Opa1. The binding pose of hyperoside within the Opa1 structure (PDB ID: 8EFS) was predicted by molecular docking. Binding affinities were estimated using AutoDock Vina. Protein-ligand interactions and docking conformations were visualized with Discovery Studio Visualizer. This docking analysis provides structural insight into the potential interaction between hyperoside and Opa1, supporting its involvement in Opa1-associated mitochondrial pathways.

**
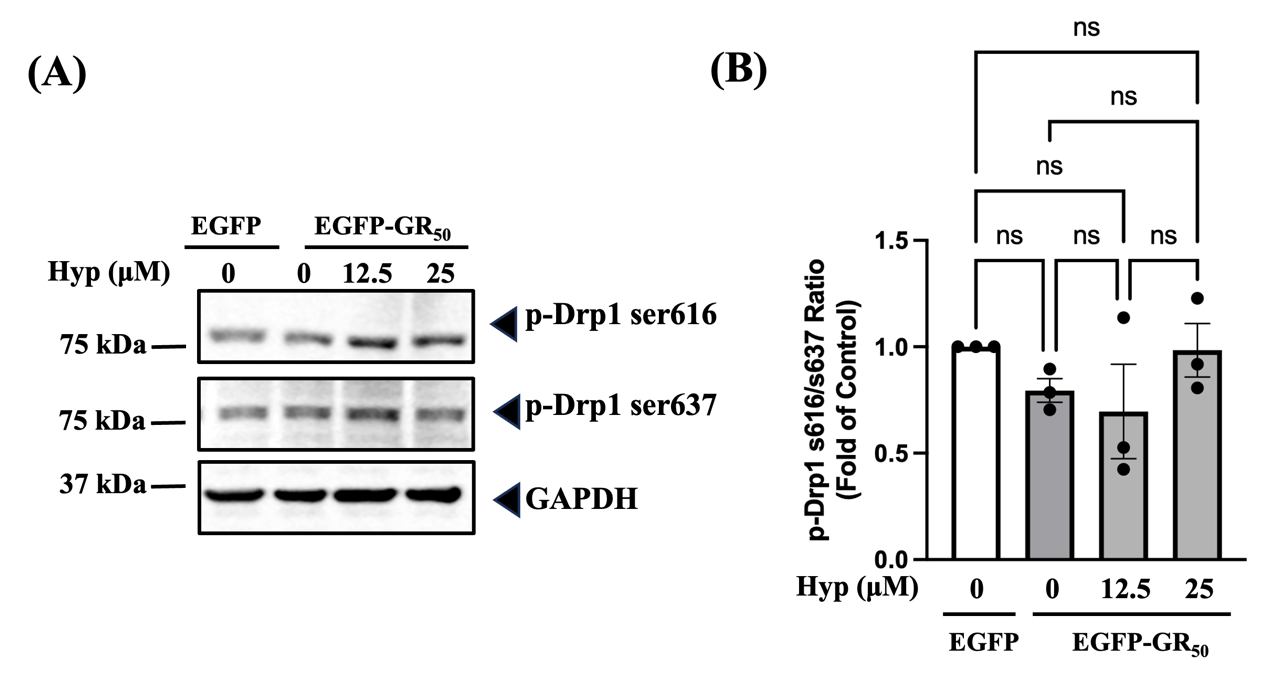
**

**Supplementary Figure 4. Hyperoside does not significantly affect Drp1 phosphorylation in EGFP-GR_50_-expressing NSC34 cells.** (A-B) Western blot analysis of p-Drp1 (Ser616) and p-Drp1 (Ser637) protein levels in EGFP-GR_50_-expressing NSC34 cells following hyperoside treatment. Data are presented as mean ± SEM and were analyzed using one-way ANOVA followed by Tukey’s multiple comparisons test. n = 3 independent biological replicates.


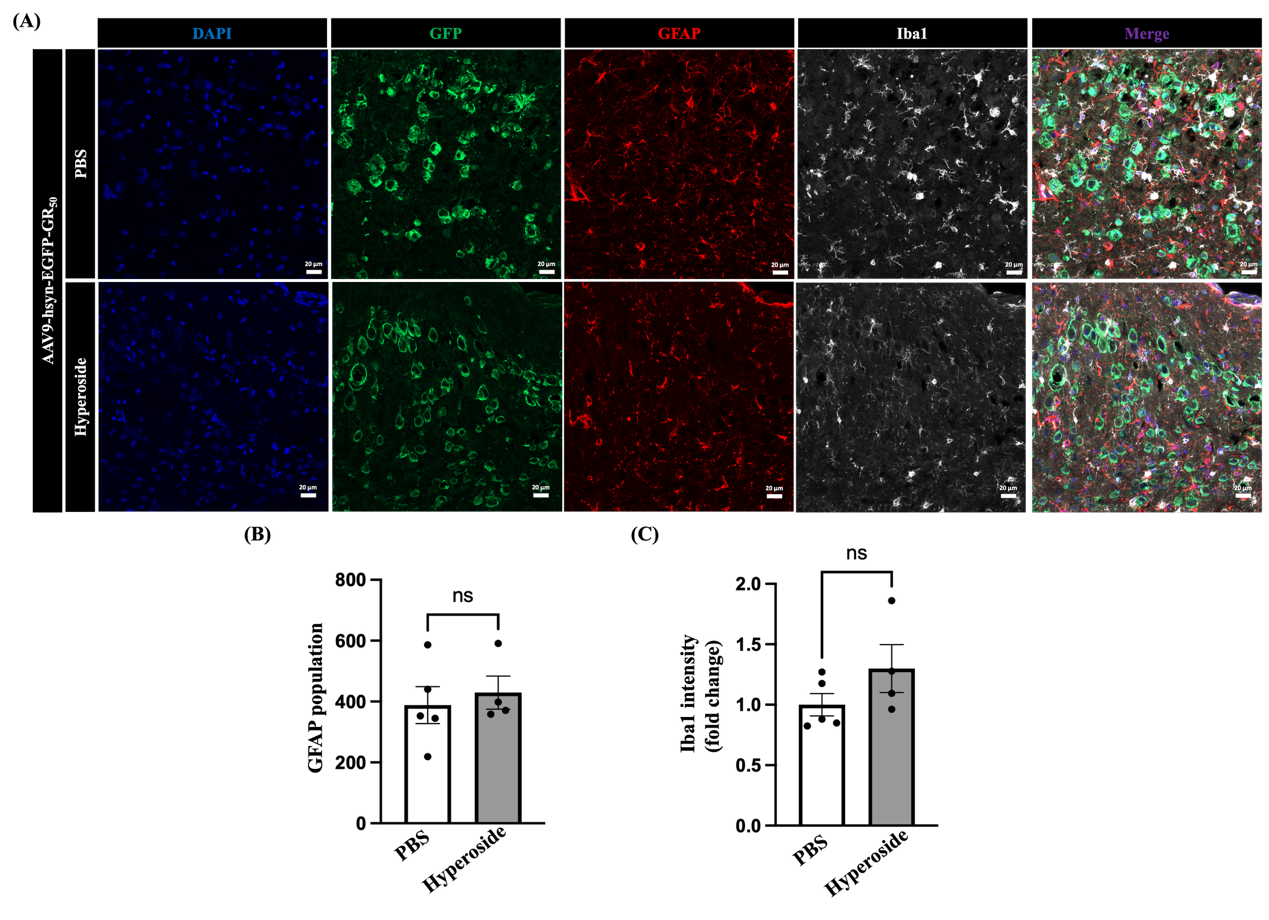


**Supplementary Figure 5. Hyperoside does not significantly alter astrocyte or microglial responses in the AAV9-hSyn-EGFP-GR_50_ model.** (A) Representative immunofluorescence images of motor cortex sections from mice injected with AAV9-hSyn-EGFP-GR_50_ and treated with PBS or hyperoside. Brain sections were stained with DAPI (blue), GFP (green), GFAP (red), and Iba1 (white). Merged images are shown in the right panel. (B) Quantification of GFAP-positive astrocytes and (C) Iba1 immunoreactivity revealed no significant differences between PBS- and hyperoside-treated groups under EGFP-GR_50_ expression. Data are presented as mean ± SEM and analyzed using an unpaired two-tailed t-test (n = 5 animals per PBS control group and n=4 animals per hyperoside group).
