# Supplementary material for: Hyperoside protects against poly-GR-mediated neurodegeneration via regulation of mitochondrial fission and oxidative stress in C9orf72-associated ALS
Source: Chin Med. 2026 Jun 4;21:161. doi: 10.1186/s13020-026-01433-w (PMC13235191; doi:10.1186/s13020-026-01433-w)

**Figure 2 (E) and (I) show the whole blot after cutting the membrane at the molecular** **weights 75 kDa, 50 kDa, and 37 kDa for Drp1 (80 kDa), OPA1 (80-100 kDa), Nrf2 (110 kDa), HO-1 (28 kDa), GPx4 (22 kDa), and GAPDH (37 kDa)**


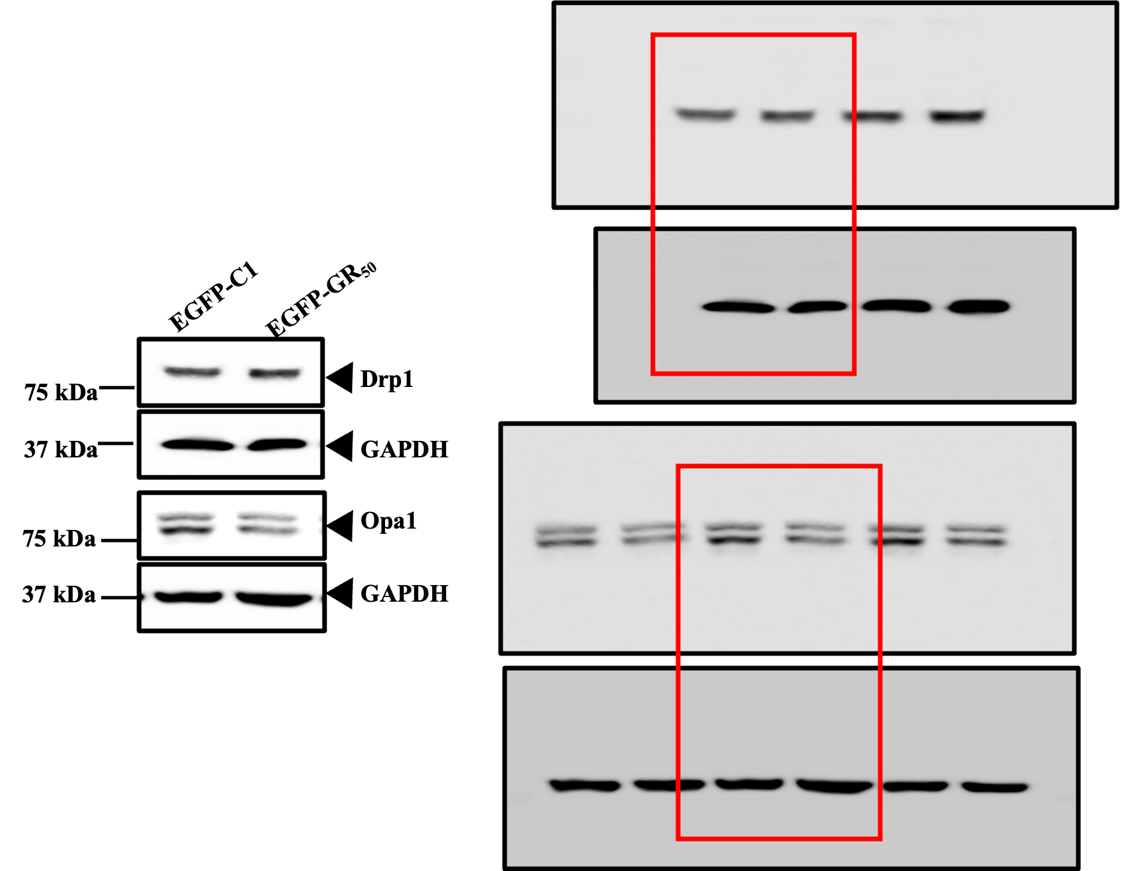


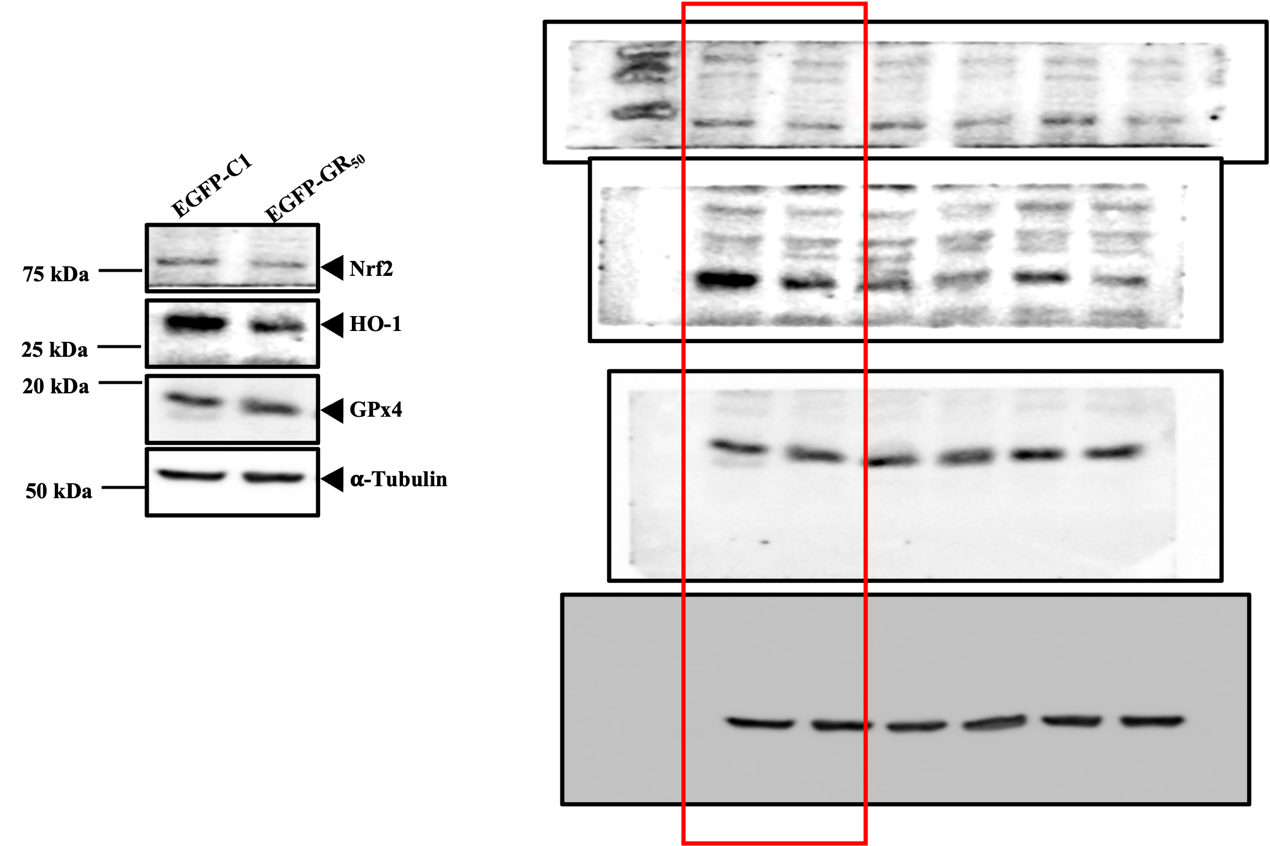


**Figure 3 (C) shows the whole blot after cutting the membrane at** **molecular weights 75 kDa and 37 kDa for r Drp1 (80 kDa), OPA1 (80-100 kDa), and GAPDH** **(37 kDa)**


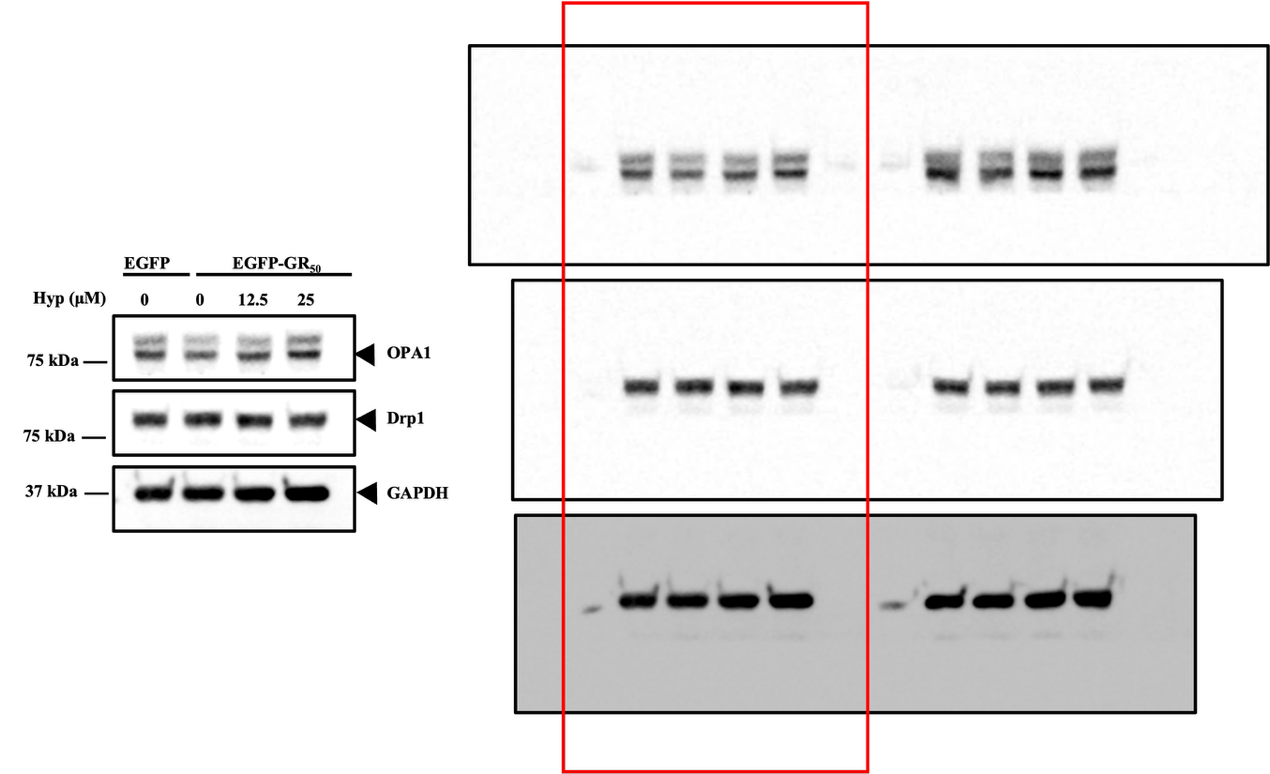


**Figure 4 (C) and (G) show the whole blot after cutting the membrane at the molecular** **weights 75 kDa, 50 kDa, and 37 kDa for Nrf2 (110 kDa), HO-1 (28 kDa), GPx4 (22 kDa), and GAPDH (37 kDa)**

**
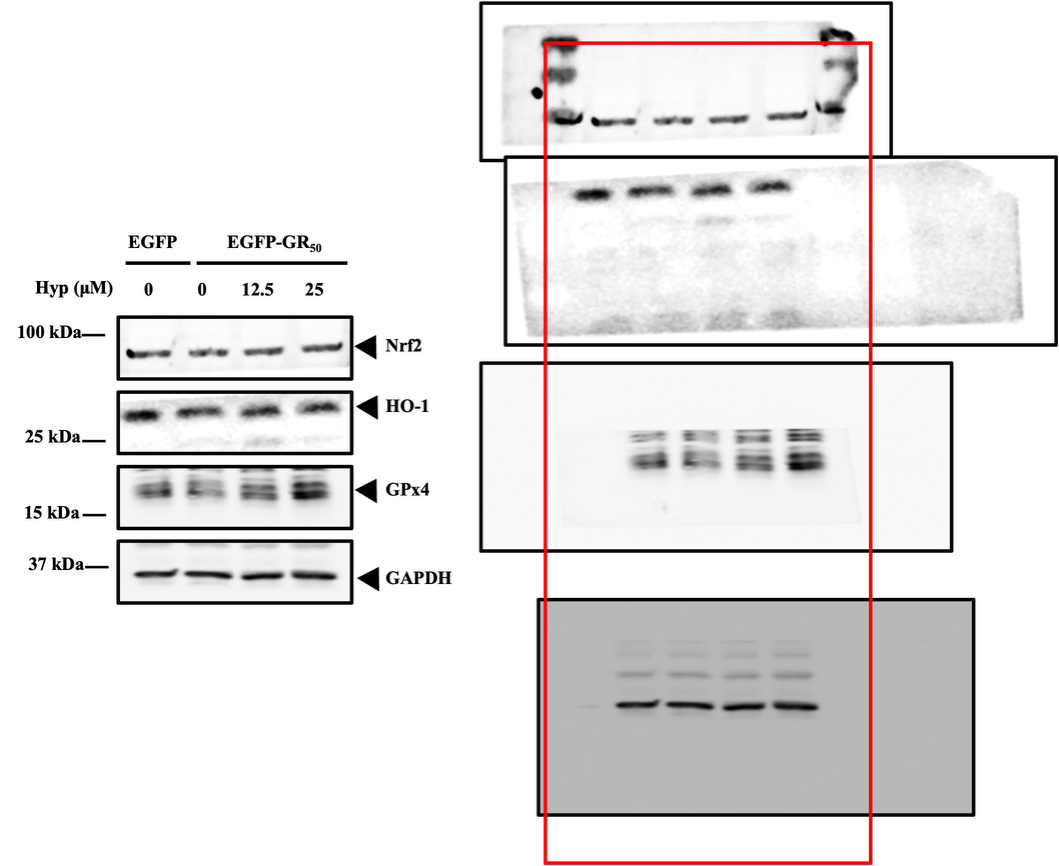
**

**
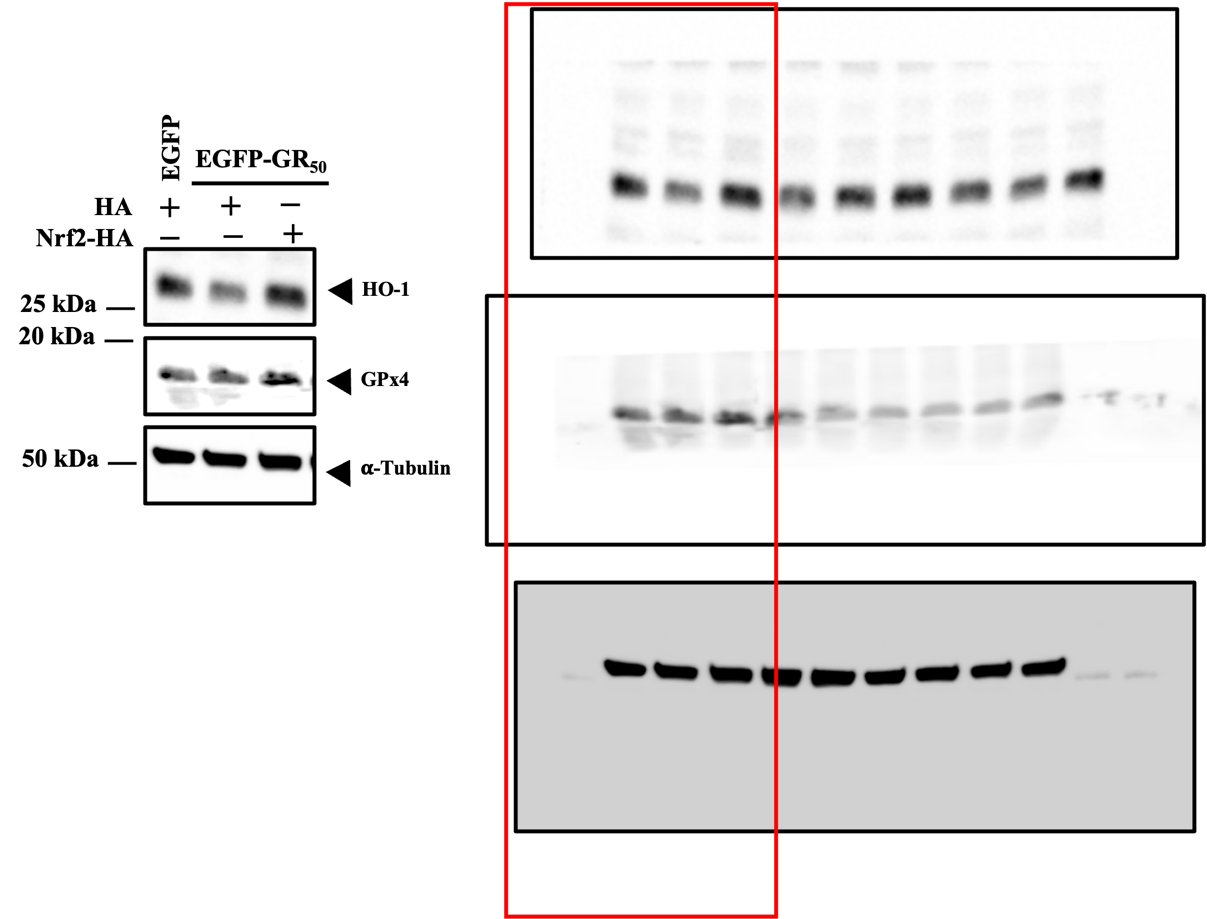
**

**Figure 6 (A) and (C) show the whole blot after cutting the membrane at the molecular** **weights, 50 kDa, 37 kDa, 25 kDa, and 20 kDa for C-caspase 3 (17 kDa), Bax (21 kDa), Bcl-2 (26 kDa), β-actin (43 kDa), 𝛼-Tubulin (55 kDa) and GAPDH (37 kDa)**


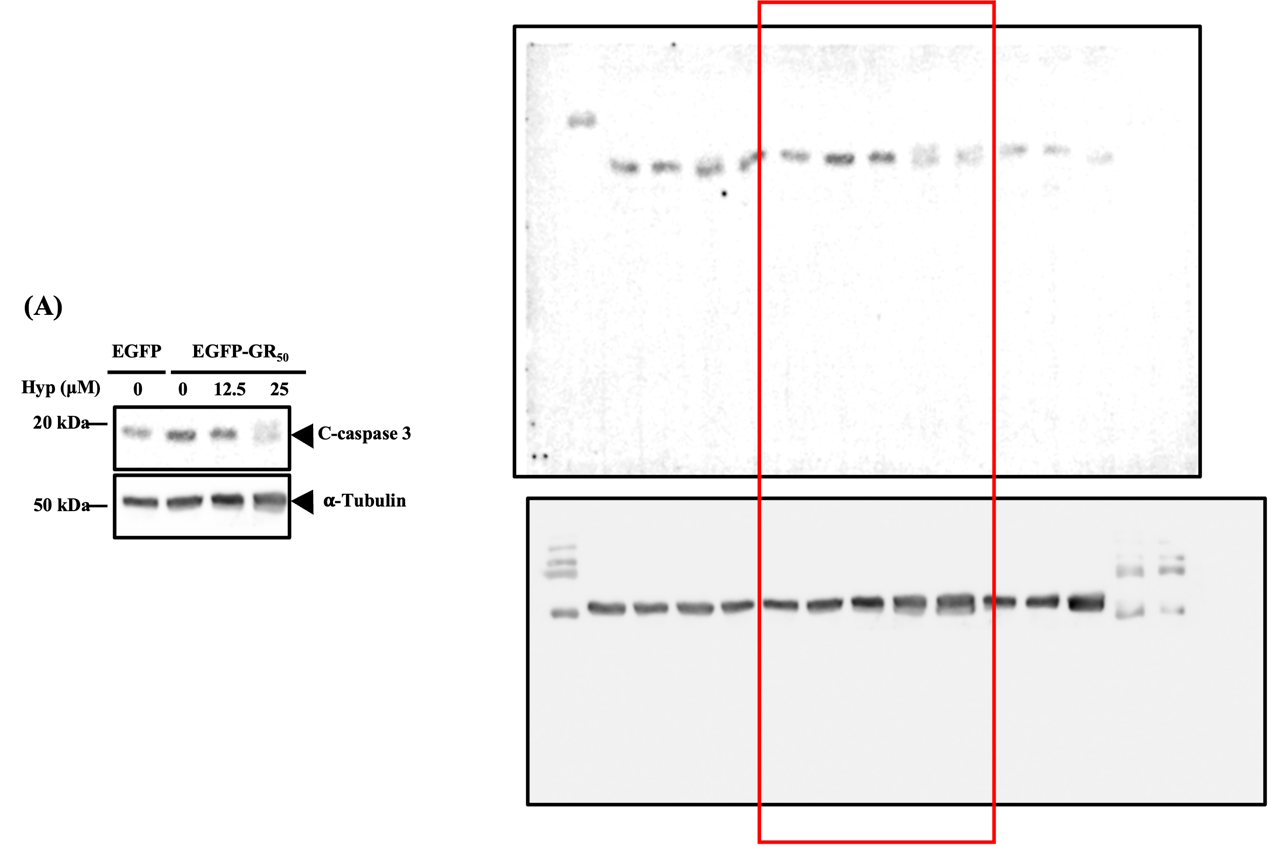


**(C)**


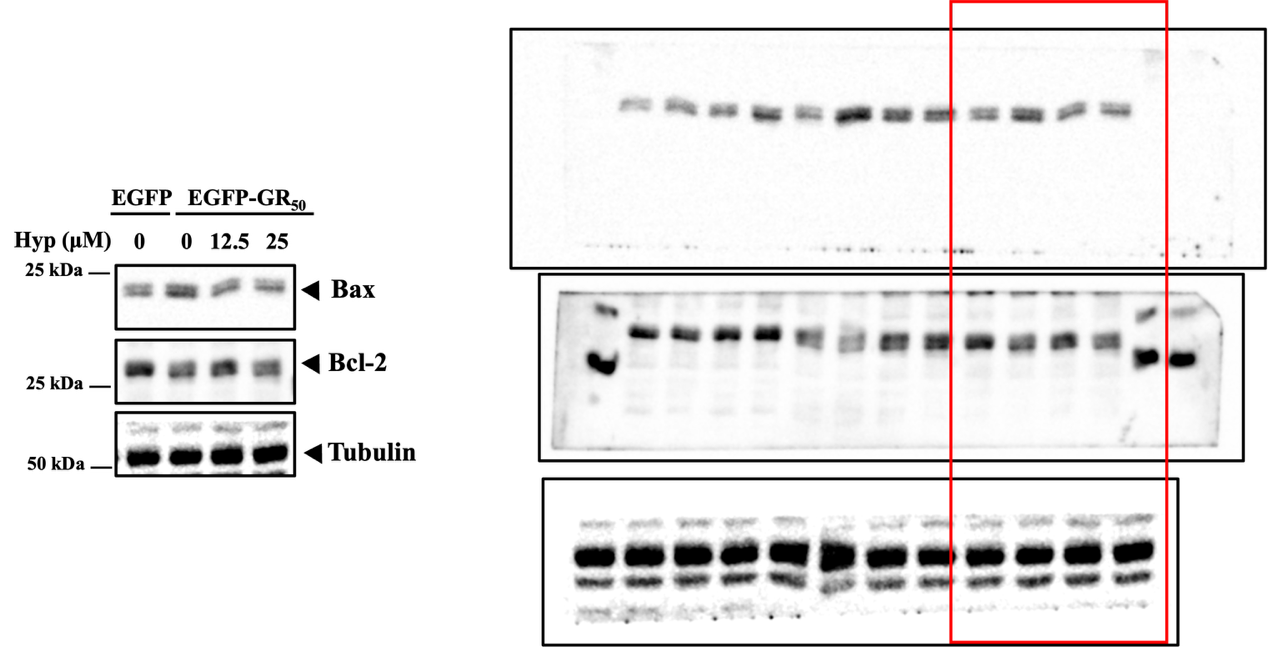

Supplement: Supplementary file 2 — Supplementary material 2. [file 13020_2026_1433_MOESM2_ESM.docx]
